# *In situ* Studies of Electrochemical Energy Conversion and Storage Technologies: From Materials, Intermediates, and Products to Surroundings

**DOI:** 10.1007/s40820-025-02014-6

**Published:** 2026-01-05

**Authors:** Xing Chen, Yu-Lin Sun, Xiu-Mei Lin, Jin-Chao Dong, Jian-Feng Li

**Affiliations:** 1 College of Energy, State Key Laboratory of Physical Chemistry of Solid Surfaces, iChEM, College of Chemistry and Chemical Engineering, College of Materials, School of Life Sciences, College of Physical Science and Technology, and Discipline of Intelligent Instrument and Equipment, Xiamen University, Xiamen, 361005 People’s Republic of China; 2 College of Chemistry, Chemical Engineering and Environment, Fujian Province University Key Laboratory of Pollution Monitoring and Control, Minnan Normal University, Zhangzhou, 363000 People’s Republic of China; 3 Innovation Laboratory for Sciences and Technologies of Energy Materials of Fujian Province (IKKEM), Xiamen, 361005 People’s Republic of China

**Keywords:** *In situ* studies, Electrocatalysis, Lithium batteries, Reaction mechanisms, Structure-performance relationships

## Abstract

Escalating global energy demands and climate urgency necessitate advanced electrochemical energy conversion and storage technologies (EECSTs) like electrocatalysis and rechargeable batteries. Improving their performance relies on elucidating reaction mechanisms and structure-performance relationships via *in situ* studies. This review summarizes recent *in situ* studies of EECSTs through a variety of advanced characterization techniques aiming at mapping reaction pathways for the rational design of overall high-performance reaction systems. We outline the principles, capabilities, advantages, and limitations of various *in situ* techniques. Their applications in *in situ* studies of fuel cells, water/CO_2_ electrolysis, and lithium batteries are highlighted with representative examples. These studies enable dynamic tracking of chemical and structural evolution of overall reaction systems, including materials, intermediates, products, and surroundings during operation, providing insights critical to rational system design. Future advancements will involve integrating multimodal *in situ/operando* approaches with artificial intelligence to enable real-time monitoring at practical scales. Such integration promises precise mechanistic insights and robust structure-performance correlations, ultimately accelerating the development of high-performance EECSTs aligned with sustainability and market requirements.

## Highlights

- An overview of the principles, capabilities, advantages, and limitations of various advanced *in situ* characterization techniques is provided.
- *In situ* studies of fuel cells, water electrolysis, CO_2_ reduction reaction, and lithium batteries are reviewed across multiple scales, from materials to surroundings.
- Challenges and prospects of *in situ* studies of electrochemical energy conversion and storage technologies are proposed.

## Graphical abstract

**Figure:**
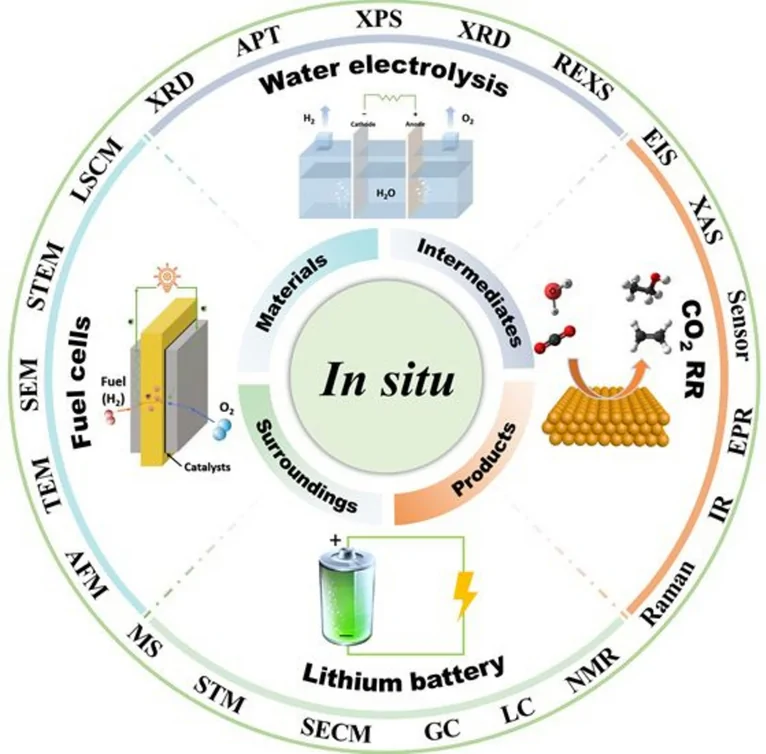

## Introduction

Rising global energy demands, fossil fuel depletion, and environmental crises [1–3] urgently call for sustainable electrochemical energy conversion and storage technologies (EECSTs), including fuel cells [4–6], water electrolysis [7, 8], CO_2_ reduction reaction (CO_2_RR) [9–11], and lithium batteries [12–16]. Enhancing their performance requires rational design of overall reaction systems, including components such as materials, intermediates, products, and surroundings based on mechanistic understanding, particularly the dynamic chemical and structural evolution of these components under operating conditions. Conventional electrochemical methods lack the spatiotemporal resolution to capture transient species or localized reactions at the molecular/atomic level. In contrast, modern advanced *in situ* characterization techniques enable real-time observation of dynamic changes without extracting samples or interrupting reactions, thereby providing more accurate and reliable insights into reaction pathways.

**Fig. 1:**
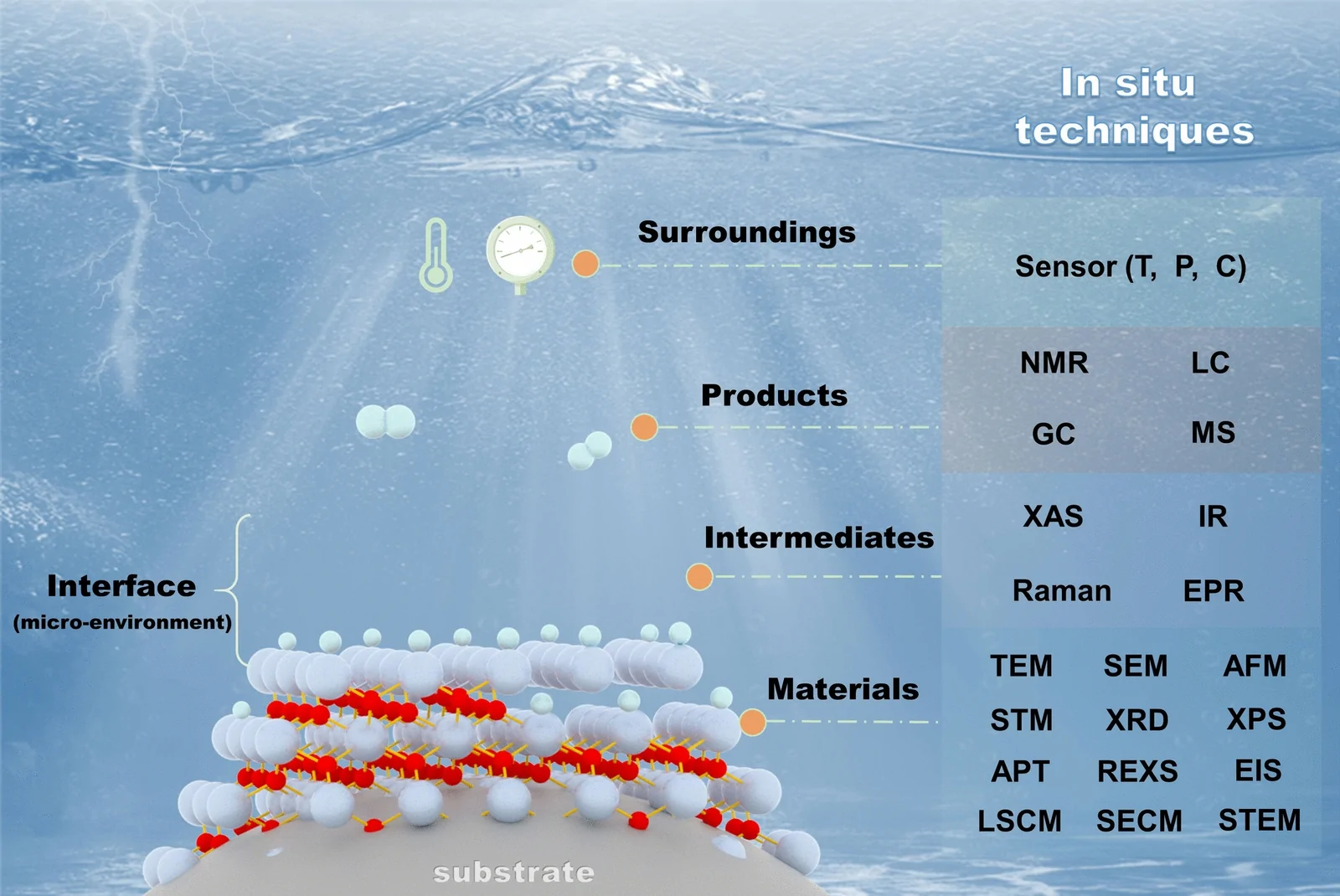
Schematic illustration of the probing species‒*in situ* characterization techniques dependence for tracking dynamic electrochemical energy conversion and storage reaction processes. Based on probing depth and target species, these methods can be categorized into four groups: materials, intermediates, products, and surroundings

Current advanced *in situ* characterization techniques encompass electron microscopy (transmission electron microscope (TEM), scanning electron microscope (SEM), and scanning transmission electron microscopy (STEM)), optical microscopy (laser scanning confocal microscopy (LSCM)), probe characterization techniques (atomic force microscope (AFM), scanning tunneling microscope (STM), scanning electrochemical microscopy (SECM), and atomic probe tomography (APT)), X-ray characterization techniques (X-ray photoelectron spectroscopy (XPS), X-ray diffraction (XRD), resonant elastic X-ray scattering (REXS) and X-ray absorption spectroscopy (XAS)), infrared spectroscopy (IR), Raman spectroscopy (Raman), electrochemical impedance spectroscopy (EIS), electron paramagnetic resonance (EPR), nuclear magnetic resonance (NMR), chromatographic techniques (liquid chromatography (LC) and gas chromatography (GC)), mass spectrometry (MS), and sensor, etc. Each technique operates on distinct principles and probes specific energy levels, providing unique spatial and temporal insights. Based on probing depth and target species, these methods can be categorized into four groups: TEM, SEM, STEM, LSCM, AFM, STM, SECM, APT, XPS, XRD, REXS, and EIS for materials characterization; XAS, IR, Raman, and EPR for intermediate species analysis; NMR, LC, GC, and MS for product detection; and sensors for monitoring the reaction surroundings (Fig. 1).

Although some excellent reviews comprehensively cover *in situ* studies of EECSTs [17–20], focusing on how electrode materials influence reaction mechanisms, they reflect the earlier view that electrochemical performance was predominantly determined by electrode composition and structure. Recently, however, growing evidence highlights the critical role of reaction surroundings (including the interfacial micro-environment), which synergistically affects intermediates, products, and reaction pathways, thereby collectively shaping reaction mechanisms [21]. A comprehensive mechanistic understanding thus requires dynamic, precise, and reliable information on materials, intermediates, products, and surroundings. Understanding the complex interactions among them, particularly the dynamic processes at the electrode–electrolyte interface, is essential.

This review surveys recent advances in *in situ* multi-techniques for EECSTs, focusing on the principles, capabilities, and limitations of various *in situ* techniques, categorized by their probing targets from a “multi-scale” perspective (ranging from material structures, intermediates, products, to device environments). It comprehensively covers several key energy conversion and storage systems, such as oxygen and hydrogen reactions in fuel cells (oxygen reduction reaction (ORR), hydrogen oxidation reaction (HOR)), water electrolysis (hydrogen evolution reaction (HER), oxygen evolution reaction (OER)), CO_2_RR, and lithium batteries (Li-ion, Li–S, and Li-O_2_). Specifically, a dedicated discussion on the emerging role of artificial intelligence and multimodal data integration. Moreover, it puts forward the current challenges and future development directions in this field. A comparison between other reviews and ours in the research scope, technique coverage, and system applications is summarized and presented in Table 1 to show the novelty of our review.

**Table 1:** Comparison between recent related works and this review

References	Research Scope	Technique Coverage	System Applications
[17]	*In situ* spectroscopy for mechanistic studies in LIBs, SCs, water splitting, and small-molecule oxidation	FT-IR (ATR-FTIR, SNIFTIRS), Raman (SERS, SHINERS), XPS (APXPS), XAS (XANES/EXAFS) for real-time monitoring of electrochemical interfaces and reactions	SEI formation in LIBs;
			Charge storage in SCs;
			Intermediate detection in HER/OER;
			Pathway analysis in oxidation reactions
[18]	*In situ* monitoring of structural, electronic, and surface changes in heterogeneous photocatalysis under real reaction conditions	TEM, XRD, XAS, SI-XPS, SPM (KPFM/SPVM), PL, EPR, UV–Vis, FT-IR (DRIFTS/ATR), Raman (SERS/TERS) techniques for probing photophysical/photochemical processes	Photocatalytic systems: water splitting (H_2_/O_2_ evolution), CO_2_ photoreduction, organic pollutant degradation, semiconductor-based photocatalysts (e.g., TiO_2_, MOFs)
[19]	*In situ* probing of active sites and mechanisms of ORR in fuel cells and metal-O_2_ batteries, focusing on Pt-based, M–N-C, and oxide catalysts	XRD, XAS, Raman, FT-IR, TEM, AFM, SECM, ETS, Mössbauer spectroscopy. Each technique’s principles, strengths, and detection capabilities are detailed	Monitoring catalyst evolution, intermediates, products, and anion adsorption on fuel cells, metal-O₂/air batteries (Li-O_2_, Na-O_2_, Zn-O_2_), Pt-based/M–N-C/oxide catalyst systems
[20]	*In situ* electrochemical characterization for energy conversion systems, focusing on real-time monitoring of reaction mechanisms via spectral, spatial, and optical techniques	Spectral (IR, UV–vis, XRD, NMR, Raman, XPS, etc.), spatial (OCT, SECM, TEM, AFM, confocal, etc.), and optical sensing techniques (TIR, SPR, WM)	Energy conversion devices: fuel cells (PEMFC, SOFC), electrolyzers (water splitting, CO_2_ electrolysis),
			Energy storage devices: Li-ion, Na-ion, K-ion, flow batteries, metal-air batteries (Zn-air, Li-air)
This review	*In situ* elucidating reaction mechanisms and structure-performance relationships for electrochemical energy conversion and storage: from materials, intermediates, and products to surroundings	TEM, SEM, STEM, LSCM, AFM, STM, SECM, APT, XPS, XRD, REXS, and EIS for characterization of materials; XAS, IR, Raman, and EPR for probing of intermediate species; NMR, LC, GC, and MS for detection of products; and sensors for sensing of the reaction environment (surroundings)	Energy conversion devices: ORR/HOR in fuel cells; HER/OER in water splitting; CO_2_RR;
			Energy storage devices: Li-ion, Li–S, Li-O_2_ batteries

This table systematically compares the characteristics of various reviews from three dimensions: research scope, technique coverage and system applications

## *In situ* Characterization Techniques

This section divides various advanced *in situ* characterization techniques into four categories based on their probing depth and species (materials, intermediates, products, and surroundings). Each technique’s basic principles, obtainable information, advantages, and limitations are discussed in detail to guide their appropriate application.

### Characterization of Materials

Electrode materials, as the core component of energy conversion and storage systems, greatly determine the overall electrochemical performance. *In situ* characterization techniques, such as TEM, SEM, STEM, LSCM, AFM, STM, SECM, APT, XPS, XRD, REXS, and EIS, provide atomic-level structural and electronic state information of electrodes under actual working conditions, which enable researchers to understand how active sites participate in reactions and how they evolve during the reaction processes. This information is crucial for the design and development of more efficient and stable electrode materials to improve the overall electrochemical performance.

#### Electron Microscopy

**Fig. 2:**
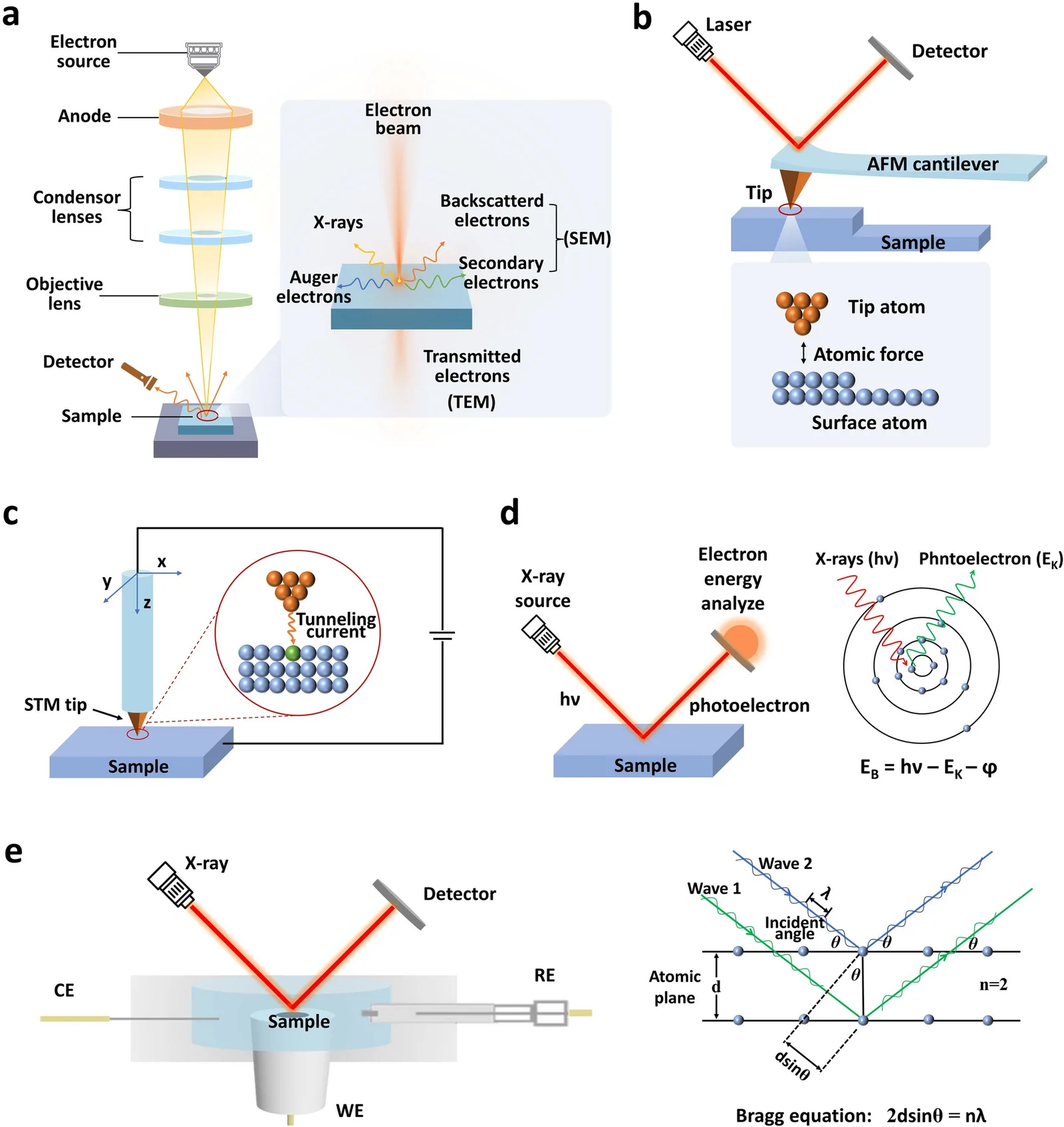
Schematic of the basic principles and instrumentations of various *in situ* techniques for the characterization of electrode materials: **a** SEM/TEM, **b** AFM, **c** STM, **d** XPS, **e** XRD

Electron microscopy, including TEM, SEM, and STEM, is a powerful tool for high-resolution imaging of samples. TEM [22, 23] uses a high-energy electron beam to penetrate thin samples, providing detailed information about the crystal structure, lattice parameters, and defects within materials (Fig. 2a). It is particularly useful for studying the microstructure of electrode materials and observing structural changes during electrochemical reaction processes. SEM [24] scans a focused electron beam across the sample surface, generating signals such as secondary and backscattered electrons to create high-resolution topographical images and compositional information. It offers a larger field of view and is effective for analyzing surface features of electrode materials.

*In situ* TEM provides atomic-level structural information and allows for the observation of internal material dynamics and active site evolution during electrochemical processes. It can simulate realistic conditions by supporting experiments under varying temperatures, pressures, atmospheres, or electric fields. However, it has limitations such as complex sample preparation, potential damage to beam-sensitive samples, and the high-vacuum environment, which may not fully replicate the actual electrochemical reaction environment. Environmental transmission electron microscopy (ETEM) overcomes the ultra-high-vacuum constraints of conventional TEM by permitting a controlled gas environment (typically on the order of pascals to kilopascals) in the sample region. This is achieved through a differential pumping system that maintains the electron gun and column sections under high vacuum, thereby preserving both the electron source and image quality. Liquid-cell transmission electron microscopy (Liquid-Cell TEM) is an advanced imaging technique that enables direct, *in situ* observation of dynamic processes in liquid environments (such as aqueous solutions), within the high-vacuum column of a TEM. By employing specialized microfluidic cells to enclose liquid samples, this method facilitates real-time monitoring of liquid-phase reactions with resolution capable of reaching the atomic scale. ETEM and Liquid-Cell TEM, from a tool for static, high-vacuum characterization, into a powerful nanoscale laboratory. These techniques allow real-time visualization of atomic and molecular dynamics in realistic environments, greatly advancing the understanding of fundamental processes in heterogeneous catalysis, materials growth, and energy storage and conversion. As such, they provide a critical link between atomic-scale structure and macroscopic properties of materials.

*In situ* SEM is primarily used to observe morphological changes in electrode materials during electrochemical reaction processes. It can produce high-magnification images and offers a greater depth of field, enabling the visualization of three-dimensional sample structures. When combined with an energy dispersive spectrometer (EDS), it provides detailed information about the composition and distribution of elements on the surface of electrode materials. However, *in situ* SEM faces challenges such as lower resolution compared to TEM, limited observation of internal structures, and the need for special sample preparation and environmental control for liquid-containing samples. STEM integrates the imaging principles of SEM with the high-resolution capabilities of TEM. In STEM, a finely focused electron probe is raster-scanned across a thin specimen. Unlike SEM, which primarily detects secondary or backscattered electrons from the surface, STEM collects electrons transmitted through the sample, thereby revealing its internal structure [25]. A key advantage of STEM is its ability to correlate structural information at sub-ångström resolution with spatially resolved elemental composition and chemical bonding states.

#### Optical Microscopy

LSCM employs a laser point source, a pinhole-guided point detector, and point-scanning technology to acquire high-resolution optical images [26]. Its core principle is confocal imaging, which ensures that the light source, the sampled point, and the detector lie at conjugate focal points, thereby effectively eliminating out-of-focus light and significantly improving image clarity. LSCM is widely used in energy research to visualize spatial distribution and morphological evolution, effectively complementing molecular-level insights from spectroscopic techniques such as Fourier transform infrared spectroscopy (FT-IR) and Raman spectroscopy. It has been most extensively applied to observe lithium dendrite growth kinetics and analyze structural evolution and failure mechanisms in electrode materials. Leveraging high spatial resolution and 3D imaging capabilities, LSCM serves as an indispensable tool for characterizing micro- and nanoscale morphological and structural changes in energy materials. Its integration with spectroscopic methods provides a comprehensive view of electrochemical processes from both morphological and chemical perspectives, greatly advancing the development of next-generation high-performance and safe energy devices. However, due to the scattering and absorption of light in the sample, the effective observation depth of LSCM is usually limited to within 100 µm. For very thick or opaque samples, sectioning is required.

#### Probe Characterization Techniques

Probe characterization techniques utilize a probe to interact with a sample, obtaining information about its properties. These techniques provide high spatial resolution, surface sensitivity, versatility, and functional imaging modes, offering detailed information about the structure, composition, and behavior of materials at the atomic, and nanoscales. Typical techniques include AFM, STM, SECM, and APT.

AFM works by using a microcantilever with a sharp tip to scan the surface of a sample (Fig. 2b). As the tip approaches the sample surface, an interaction force between the tip and the sample causes the microcantilever to deform or vibrate. A laser beam is reflected off the back of the microcantilever and onto a photoelectric detector [27]. By detecting the changes in the position of the reflected laser beam, the deformation or vibration of the microcantilever can be measured, which reflects the interaction force between the tip and the sample. AFM provides high-resolution imaging, enabling detailed observation of surface morphology and structure at the nanoscale or atomic level. STM operates based on the quantum tunneling effect, in which a very fine metal probe tip is brought extremely close to the sample surface (typically within a few atomic distances). When a small voltage is applied between the tip and the sample, electrons can tunnel through the gap between them, creating a tunneling current (Fig. 2c) [28]. The magnitude of this current is highly sensitive to the distance between the tip and the sample. By precisely controlling the movement of the tip across the sample surface and measuring the changes in the tunneling current, the surface structure and defects of the sample can be visualized at the atomic scale. STM provides atomic-level resolution imaging of the sample surface, enabling detailed observation of the surface structure and defects of electrode materials. However, STM requires the sample to be conductive and have an ultra-flat surface, limiting its application to certain materials and necessitating special sample preparation procedures. The presence of liquid in *in situ* experiments may interfere with the probe-sample interaction and affect imaging quality, and STM has a relatively slow imaging speed, which may limit its ability to capture fast-dynamic processes in some electrochemical reactions.

SECM is a prominent scanning probe technique that employs an ultramicroelectrode as a scanning probe. The probe is positioned in proximity (typically within 1 μm) to the surface of a sample immersed in an electrolyte solution. By measuring the faradaic current between the probe and the sample, SECM enables visualization of local chemical and electrochemical activity with high spatial resolution [29]. A key advantage of SECM is its ability to simultaneously provide topographic features and quantitative chemical/electrochemical information at micro- to nanoscale resolutions. However, the resolution is primarily determined by the probe size and the probe-sample distance. Achieving nanoscale resolution remains challenging, as it requires both the fabrication of nanometer-scale probes and precise control of the separation distance. APT combines field evaporation and time-of-flight mass spectrometry to provide three-dimensional, quantitative chemical mapping at near-atomic resolution [30]. This technique directly reveals nanoscale chemical heterogeneity, even down to atomic-scale compositional variations, enabling direct correlation between microstructural features (such as precipitates and grain boundaries) and material properties (including strength, corrosion resistance, and electrical characteristics). However, the preparation of undamaged, site-specific specimens (such as those containing particular grain boundaries or interfaces), remains challenging and time-consuming, posing a major bottleneck in APT analysis. Additional limitations include a restricted analysis volume and material-dependent constraints.

#### X-ray Characterization Techniques

X-ray characterization techniques, such as XPS, XRD, and REXS, utilize the interaction between X-rays and matter to obtain information about the structure and composition of materials. XPS is based on the photoelectric effect, where X-rays cause the emission of photoelectrons from the sample, and the kinetic energy of these photoelectrons is measured to determine the binding energy of the elements (Fig. 2d) [31–33], providing information about the elemental composition and chemical state of the sample surface. XRD is based on the diffraction of X-rays by the periodic arrangement of atoms in a crystal and follows the Bragg equation (Fig. 2e), allowing for the determination of crystal structure, lattice parameters, and phase composition [34–36]. REXS is an advanced synchrotron-based X-ray diffraction technique. By tuning the incident X-ray energy to the absorption edge (e.g., L-edge or K-edge) of a specific element, the resonant condition significantly enhances its scattering intensity [37]. This method enables sensitive detection of element-specific, orbital-dependent, and spin-related ordering phenomena in materials.

X-ray techniques provide significant advantages for *in situ* electrochemical characterization of material structures, offering non-destructive analysis that preserves the sample’s integrity during measurement. *In situ* XPS can track the changes in the surface composition and chemical state of materials under electrochemical conditions, providing insights into the mechanisms of electrochemical reactions and the stability of the material’s surface. However, its high-vacuum requirements may distort electrochemical interfaces and limit shallow penetration for bulk defect analysis. Complex sample preparation and time-consuming measurements further constrain its utility. *In situ* XRD can provide real-time information about the structural changes of materials during electrochemical reactions, such as phase transformations and lattice parameter variations, which is crucial for understanding the relationship between the material’s structure and its electrochemical performance. However, the strong attenuation of primary and diffracted beams within the electrolyte solution can reduce the intensity of X-ray signals, making *in situ* XRD difficult to obtain clear diffraction patterns. To mitigate this, a thin layer of electrolyte solution is often used, but this can limit the electrochemical measurements and may not fully replicate the actual reaction environment. Additionally, *in situ* XRD may struggle to detect amorphous materials or small structural changes, as these do not produce distinct diffraction peaks. The key advantage of *in situ* REXS lies in its ability to tune the X-ray energy to the absorption edges of different elements, enabling element-specific probing of their individual roles in complex materials. This facilitates the "decomposition" of each element’s contribution to the ordered state. However, REXS imposes stringent sample requirements, typically high-quality single crystals or well-ordered periodic structures, and is subject to limitations such as radiation damage and limited temporal resolution.

#### Electrochemical Impedance Spectroscopy (EIS)

EIS is a powerful frequency-domain technique that probes complex electrochemical processes by measuring a system’s response to low-amplitude alternating current signals across a range of frequencies. It provides multi-scale dynamic information from rapid electron transport to slow ion diffusion, serving as a critical link between macroscopic properties and microscopic interfacial phenomena [38]. To interpret EIS data, equivalent circuit models are used to simulate electrochemical systems. Through numerical fitting, the circuit that best matches the experimental spectrum is identified, enabling the quantification of key physical parameters—a core yet challenging aspect of EIS analysis. Widely applied in studies of electrode–electrolyte interfaces, EIS can, for example, distinguish performance degradation mechanisms in batteries (e.g., active material loss, SEI growth, lithium inventory reduction) and monitor the evolution of interfacial films. However, EIS requires strict measurement conditions and system stability, and it only reflects global averaged responses, lacking spatial resolution to distinguish local heterogeneities on the electrode.

### Probing of Intermediate Species

During the electrochemical reaction processes, the formation and evolution of intermediate species are key to understanding the reaction mechanism. These intermediate species are typically short-lived and highly reactive, and their chemical structures can vary significantly depending on the involved specific reaction and electrode materials. They may exist as ions, radicals, or molecules with unique structural features such as specific bond lengths, bond angles, and electronic configurations, which can be probed by several techniques.

#### X-ray Absorption Spectroscopy (XAS)

**Fig. 3:**
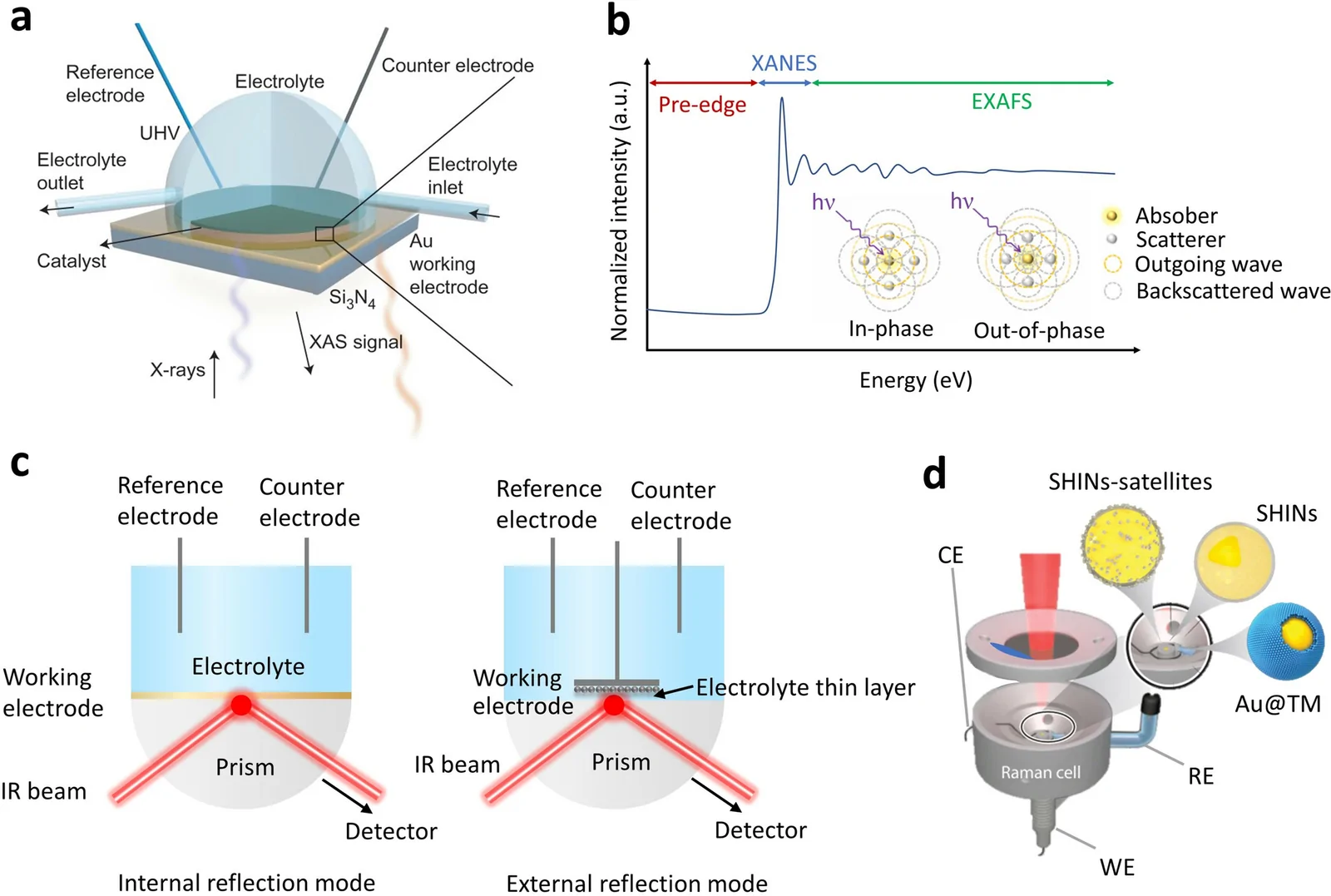
Schematic of the basic principles and instrumentation of various characterization techniques for capturing intermediates. **a** Schematic illustration of the *in situ* liquid-cell experimental setup. Reproduced with permission from Ref. [39]. Copyright 2017, Springer Nature. **b** An XAS spectrum, showing that it consists of three parts: the pre-edge (red), the XANES (cyan), and the EXAFS (green). The insets show the interference between photoelectrons and backscattered electrons in the EXAFS. Reproduced with permission from Ref. [40]. Copyright 2021, Elsevier B.V. **c** FT-IR in internal and external reflection modes. Reproduced with permission from Ref. [44]. Copyright 2021, Elsevier B.V. Internal reflection mode is widely used in electrochemical studies. Infrared light undergoes total internal reflection within an ATR crystal (e.g., ZnSe, Ge, or diamond), producing an evanescent wave that penetrates the sample near the crystal surface to a depth of several micrometers. By depositing the working electrode directly onto the ATR crystal, ultra-high-sensitivity detection at the electrode/electrolyte interface is achieved. **d** Schematic diagram of SERS applications with different nanostructures in electrocatalysis. Reproduced with permission from Ref. [46]. Copyright 2021, Annual Reviews

XAS is a powerful tool for probing the local electronic and geometric structure of intermediate species. It involves exposing a sample to a beam of X-rays with varying energies. When the energy of the incident X-rays matches the absorption edges of the elements in the sample, electrons are excited from core levels to higher energy states or unoccupied orbitals (Fig. 3a) [39]. The resulting XAS spectrum provides information about the energy levels of electrons, the oxidation states of elements, and the local atomic arrangement around the absorbing atoms. By analyzing features such as the absorption edges and the extended X-ray absorption fine structure (EXAFS) (Fig. 3b), researchers can gain insights into the electronic structure, coordination environment, and bonding properties of the intermediate species [40]. This is crucial for understanding its physical and chemical properties. *In situ* XAS offers significant advantages in characterizing electrochemical intermediate species. It provides real-time information about the electronic and structural properties of these species, allowing for the tracking of changes in oxidation states and local atomic arrangements during the reaction processes.

Notably, grazing-incidence X-ray absorption spectroscopy (GI-XAS) can confine the probe depth to the nanoscale by precisely controlling the X-ray incident angle. It can selectively probe different depth regions from the surface layer to the subsurface layer, thereby enabling specific analysis of the electronic structure and coordination environment of active sites at the electrode surface and solid–liquid interface [41]. However, *in situ* XAS also has some limitations. The experimental setup for *in situ* XAS is complex and may require specialized equipment and conditions. Additionally, the interpretation of XAS data is challenging and may require advanced theoretical models and computational methods. Furthermore, the technique may have limited temporal resolution in some cases, making it difficult to capture fast-transient intermediate species in certain electrochemical reactions.

#### Infrared Absorption Spectroscopy (IR)

IR is a technique that uses the absorption of infrared light by molecules in a sample. When a sample is exposed to infrared radiation, molecules absorb specific frequencies of light corresponding to their vibrational and rotational energy transitions, resulting in characteristic absorption bands in the infrared spectrum that correspond to the functional groups present in the molecules [42, 43]. By analyzing the position, intensity, and shape of these absorption bands, researchers can identify the functional groups and determine the molecular structure and composition of the sample. This technique offers high sensitivity for detecting trace amounts of substances, provides detailed insights into chemical structures and bonding environments, and enables real-time monitoring of chemical reactions, making it a powerful tool for studying molecular interactions.

*In situ* FT-IR (typical examples include internal reflection mode and external reflection mode) allows for real-time monitoring of chemical or electrochemical reactions, providing immediate information about the formation and evolution of intermediate species (Fig. 3c) [44]. It has high sensitivity to trace substances and offers detailed structural information about molecules, making it ideal for studying reaction mechanisms. Enhanced infrared spectroscopy, such as surface-enhanced infrared absorption spectroscopy (SEIRAS), further enhances the signal-to-noise ratio, enabling the detection of low-concentration species and providing detailed information about surface-adsorbed species. However, the experimental setup for *in situ* IR can be complex, and the presence of electrolytes may interfere with the measurements. Enhanced infrared spectroscopy may have difficulty in detecting low-frequency regions and may require advanced data interpretation methods. Additionally, sample preparation for enhanced infrared spectroscopy may introduce additional variables and complicate the experimental processes. A significant drawback of *in situ* infrared spectroscopy is its susceptibility to interference from water. Water has strong absorption in the infrared region, which can interfere with the analysis of samples containing water or that are exposed to moisture. This can affect the accuracy of the measurements and make it difficult to detect the absorption bands of the species of interest. This is usually overcome by techniques such as using a thin-layer electrochemical cell, or adopting subtraction spectroscopy (collecting a background spectrum and then subtracting the water signal). With the development of combined techniques such as synchrotron radiation infrared, and AFM-IR, their spatial resolution and functionality will be further enhanced, and there will be broader application prospects in this field in the future.

#### Raman Spectroscopy

When an incident photon interacts with a molecule, an electron transitions from a lower molecular orbital to a virtual excited state and then quickly returns to the lower molecular orbital. If the energy released by the electron during the return processes is equal to the energy difference of a certain vibrational or rotational energy level of the molecule, then this process is Raman scattering [45]. The difference between the frequency of the Raman scattered light and the frequency of the incident light is called the Raman shift, which reflects the information of molecular vibrations and rotations, and different chemical bonds and molecular structures will produce specific Raman shifts, thereby enabling the analysis and identification of substances. Moreover, enhanced Raman spectroscopy [46], including surface-enhanced Raman spectroscopy (SERS), tip-enhanced Raman spectroscopy (TERS), and shell-isolated nanoparticle-enhanced Raman spectroscopy (SHINERS), is a highly sensitive technique for detecting trace substances and studying molecular properties at interfaces (Fig. 3d). SERS enhances the Raman signal of molecules adsorbed on rough metal surfaces or nanostructures through localized surface plasmon resonance. TERS utilizes a metal nano-tip to locally enhance the electromagnetic field, achieving nanoscale spatial resolution and high-sensitivity detection. SHINERS employs shell-isolated nanoparticles, which consist of a metal core surrounded by a thin, chemically inert, and insulating shell, to enhance the Raman signal while reducing interference from the substrate and improving the applicability to various materials and surfaces. The integration of spectroscopic techniques with electrochemical measurements enables effective tracking of surface and interfacial processes during electrocatalytic reactions. In photoelectrocatalysis, a controllable light source (e.g., LED or laser) must be incorporated, with careful alignment to ensure the illumination path (typically back- or side-incident) does not interfere with the spectral detection optics.

*In situ* Raman spectroscopy allows for real-time monitoring of chemical reactions, providing immediate information about the formation and evolution of surface species. However, it has limitations such as low signal intensity for trace species and fluorescence interference. Enhanced Raman spectroscopy techniques, such as SERS and SHINERS, significantly enhance the Raman signal, enabling the detection of trace species with high sensitivity and surface selectivity. They can be applied to a wide range trace species, allowing for real-time monitoring of chemical reactions. Time-gated Raman spectroscopy (TGRS) is a time-domain filtering technique that exploits the distinct temporal characteristics of Raman and fluorescence signals. By utilizing a gated detection system, this method selectively captures the instantaneous Raman signals arriving immediately after laser excitation while suppressing delayed fluorescence emissions. However, these techniques also have limitations, including substrate dependency, stability issues, and complex data interpretation.

#### Electron Paramagnetic Resonance (EPR)

EPR, also known as electron spin resonance (ESR), is a powerful and highly specific spectroscopic technique used to detect and characterize unpaired electrons. When a sample is irradiated with electromagnetic waves of frequency ν (typically in the microwave range), resonance absorption occurs if the energy of the photons (hν) matches the energy difference ΔE between electron spin states, that is, hν = gμB₀ (where g is the g-factor, μB is the Bohr magneton, and B₀ is the external magnetic field strength) [47]. Under this condition, electrons in the lower energy state absorb energy and transition to the higher state. The instrument detects this absorption phenomenon; by sweeping the magnetic field strength and recording the corresponding absorption signals, an EPR spectrum is obtained. It not only confirms the presence of paramagnetic centers but also provides detailed information (such as identity, local environment, and concentration), through spectral parameters including the g-factor and hyperfine coupling constants. Owing to its unique sensitivity to electronic and molecular structure, EPR/ESR plays an indispensable role in a wide range of fields, from fundamental research to industrial applications. EPR spectroscopy is among the most sensitive techniques for detecting unpaired electrons, with a detection limit on the order of 10⁻^9^ to 10⁻^12^ mol. This high sensitivity enables direct identification of short-lived reaction intermediates, such as radical species. However, it is exclusively sensitive to systems with unpaired electrons and is ineffective for diamagnetic materials, where all electrons are paired, such as the vast majority of organic and biomolecules in their ground state.

### Detection of Products

The formation mechanism and characteristics of products in the energy conversion and storage processes are crucial for optimizing system performance. *In situ* spectroscopic techniques can monitor the formation of products, providing detailed information about product release kinetics and surface reactions. This information is significant for optimizing reaction conditions, improving product selectivity, and reducing side reactions.

#### Nuclear Magnetic Resonance (NMR)

**Fig. 4:**
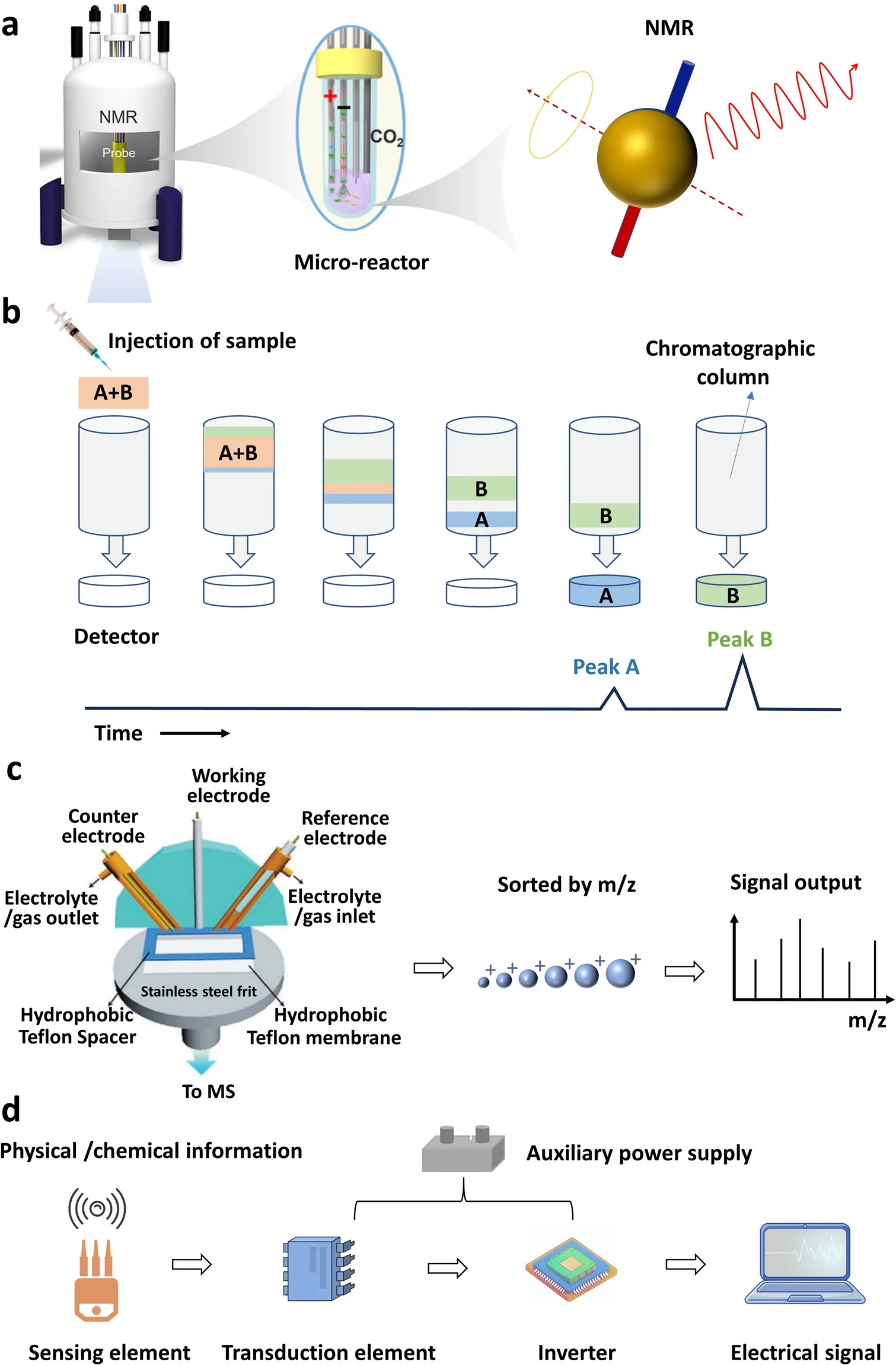
Schematic of the basic principles and instrumentation of various characterization techniques for reaction products and environments. **a** Schematic of the NMR experimental layout. Reproduced with permission from Ref. [49]. Copyright 2024, Elsevier Inc. **b** Schematic illustration of the operational mechanisms in liquid chromatography and gas chromatography. **c** Schematic of DEMS cell. Reproduced with permission from Ref. [54]. Copyright 2021, Wiley‐VCH GmbH. **d** Schematic diagram of sensor operating principles

Nuclei in a static magnetic field, when their nuclear spin quantum number is not zero, will absorb radio frequency electromagnetic waves of a specific frequency and undergo nuclear magnetic resonance (Fig. 4a) [48, 49]. The resonance frequencies of different nuclei are different. By detecting these resonance signals and their characteristics, information such as the local chemical environment of the nuclei can be obtained. By analyzing the solution or gas after the electrochemical reaction, the types and relative contents of the products produced by the reaction can be determined. In the *in situ* characterization of electrochemical products, NMR provides detailed chemical information, allowing for the identification of different chemical groups and their environments. NMR is also a non-destructive technique, meaning it does not alter the sample during measurement, making it suitable for studying samples under actual working conditions. Additionally, NMR can be applied to a wide range of samples, including liquids and solids, providing a comprehensive understanding of the processes involved. However, NMR has limited sensitivity for some nuclei, such as ^13^C, which can make it challenging to detect trace amounts of certain electrochemical products and battery interface products. The preparation of samples for NMR can be complex, especially for solid samples, which may involve dissolving the sample in a suitable solvent or preparing a solid-state NMR sample. This can introduce additional variables and complicate the experimental processes. Furthermore, the strong magnetic field required for NMR can interfere with the electrochemical reactions and the performance of the battery, affecting the accuracy and reliability of the results.

#### Chromatographic Characterization Techniques

Chromatographic techniques, including liquid chromatography (LC) and gas chromatography (GC), separate and analyze sample components based on their interactions with stationary and mobile phases (Fig. 4b). They enable accurate identification and quantification of substances in complex mixtures, making them ideal for detecting trace amounts of transient intermediates and reaction products. In LC, a liquid mobile phase carries the sample through a stationary phase, where components interact differently, leading to separation based on their varying speeds [50]. The separated components are then detected and quantified. LC specializes in analyzing non-volatile, thermally sensitive compounds like macromolecular organics and polar substances, proving valuable for tracking electrochemical intermediates and products. GC, on the other hand, uses a gas as the mobile phase and is suitable for volatile samples. The sample is vaporized and carried through the stationary phase by the gas, with components eluting at different times based on their affinities for the stationary phase [51, 52]. GC is commonly used for low-molecular-weight organic compounds and gases.

The integration of chromatography (GC or HPLC) and mass spectrometry (MS) exemplifies a highly synergistic analytical strategy [53]. Chromatography serves as a high-resolution separation tool, effectively resolving complex mixtures into individual components that are introduced sequentially into the mass spectrometer. MS then acts as a sensitive identification module, performing molecular weight determination and structural elucidation for each purified component. This combination enables accurate qualitative and quantitative analysis while overcoming critical limitations of direct injection MS, such as ion suppression, where competitive ionization suppresses signals of certain components, and spectral interference caused by overlapping ions from co-eluting species. With its high-resolution chromatography and reproducible electron ionization (EI) spectral libraries, GC–MS is the preferred method for analyzing volatile small-molecule mixtures, enabling accurate and high-throughput qualitative identification. HPLC–MS offers broad applicability and powerful tandem mass spectrometry capabilities, including multiple reaction monitoring (MRM) and high-resolution mass analysis, making it particularly suitable for the highly selective and sensitive quantification and structural characterization of polar, thermally labile, and trace-level components within complex matrices. By integrating the superior separation power of chromatography with the structural elucidation capabilities of mass spectrometry, *in situ* GC–MS and HPLC–MS hyphenated techniques have irreplaceable advantages in distinguishing isomers, providing quantitative information, and resolving complex reaction networks.

While LC excels in handling labile compounds and GC offers a rapid analysis of volatiles, both face limitations: *In situ* GC is unsuitable for non-volatile and thermally unstable substances, which may decompose or fail to vaporize. LC may have lower sensitivity for certain non-volatile substances compared to GC, and its slower analysis speed can limit its use in real-time monitoring of fast-dynamic processes. Both techniques may require long analysis times, making it challenging to capture transient intermediates in real-time. Additionally, complex sample matrices can interfere with separation and detection, affecting the accuracy. Although chromatographic methods minimize sample preparation, careful handling is essential to ensure reliable *in situ* characterization of dynamic electrochemical systems.

#### Mass Spectrometry (MS)

In MS, sample molecules are ionized into ions in the ion source, and these ions are accelerated and separated according to their mass-to-charge ratio under the action of an electric field or a magnetic field, and then detected and recorded by the detector. Ions with different mass-to-charge ratios form mass spectra, and the relative molecular mass and structure of the molecule can be determined by analyzing the mass spectra (Fig. 4c) [54]. *In situ* MS technology is usually combined with LC and GC techniques to detect reaction products in real-time, helping to confirm the reaction path and evaluate the catalytic efficiency. MS offers high sensitivity and accurate molecular mass information. It can detect trace amounts of substances and distinguish between different isotopes, aiding in the identification and quantification of specific compounds. However, MS has certain limitations. It is not suitable for non-volatile or thermally unstable compounds, as these may decompose during the ionization processes. Additionally, molecular fragmentation can occur during ionization, potentially leading to incomplete structural information. MS also requires pure samples and may not be able to distinguish between isomers or complex mixtures without prior separation. Furthermore, it demands specialized equipment and expertise for operation and data interpretation.

Differential electrochemical mass spectrometry (DEMS) is a powerful analytical technique that couples electrochemistry with mass spectrometry in real time and online [55]. It is primarily used for the qualitative and quantitative detection of volatile products or intermediates generated or consumed during electrochemical reactions. The electrochemical cell is designed with an inlet (e.g., a porous electrode or capillary) positioned in proximity to the working electrode. This configuration allows gaseous products to diffuse into the mass spectrometer via the shortest path with minimal delay. Efficient and rapid transfer of electrochemically generated species into the mass spectrometer’s vacuum system is essential for its operation. DEMS offers high sensitivity, high time resolution, and excellent qualitative and quantitative capabilities.

### Sensing of Reaction Environment

Electrochemical reactions occur under specific conditions, and environmental factors have a significant impact on their kinetics and stability. *In situ* sensing technology can monitor the pressure and temperature of the surroundings, thereby revealing how environmental factors affect the electrochemical reaction processes. Sensors usually use physical, chemical, or biological effects to convert the physical quantity to be measured (such as temperature, pressure, and concentration, etc.) into an electrical signal or other measurable signal output [56]. A sensor generally consists of four parts: a sensing element, a transduction element, an inverter, and an auxiliary power supply. The sensing element directly senses the measured quantity and outputs a physical quantity signal that has a definite relationship with the measured quantity; the transduction element converts the physical quantity signal output by the sensing element into an electrical signal; the inverter is responsible for amplifying and modulating the electrical signal output by the transduction element; the transduction element and the inverter generally also require an auxiliary power supply (Fig. 4d).

Notably, interfacial micro-environment (such as the local pH, ion concentration, and electric field) plays a critical role in energy and catalytic systems [21]. This region, situated at the phase interface (e.g., solid–liquid or solid–gas), exhibits physical and chemical properties distinct from those of the bulk phase. Traditionally, studies of electrocatalytic reactions have assumed interfacial conditions (including reactant concentration, pH, electric field, and solvent structure) to be equivalent to those in the bulk solution. However, the concept of the interfacial micro-environment challenges this assumption, highlighting that the local conditions can differ significantly from macroscopic averages. It is this unique micro-environment, rather than bulk properties, that ultimately governs reaction performance, pathway, and kinetics. Strategies such as engineering surface hydrophobicity/hydrophilicity, constructing confined architectures, and performing surface functionalization enable the precise control of the local micro-environment to steer electrocatalytic processes toward desired outcomes. Accurately quantifying the local micro-environment (such as pH, specific ion concentration, and electric field strength) at the electrode/electrolyte interface is crucial for understanding the local behavior and mechanisms of electrochemical reactions. The following Table 2 summarizes the typical operating ranges and characteristics of several key micro-environment probe techniques.

**Table 2:** Summary of local micro-environment probe techniques

	Probe technique	Principle	Spatial resolution	Indicative range & notes
Local pH	Fiber-Optic pH Sensors	pH-sensitive dye at fiber tip	1 µm – 100 µm	pH 2 – 12 (depends on dye)
		Fluorescence intensity/lifetime changes with pH		Fast response, minimal electrical interference
				Ideal for biological samples
	SECM	Uses an ultramicroelectrode (UME) to measure H⁺-dependent Faradaic current (e.g., quinone reduction)	10 nm – 1 µm	Full pH range (depends on mediator)
				Quantitative mapping, can be coupled with topography
	Nano-/Micro-pipettes (pH-sensing)	Pipette filled with pH-selective liquid membrane or functionalized at the tip	10 nm – 1 µm	pH 2 – 12
				Can be integrated with SICM for simultaneous topography
Local Ion Concentration	Scanning Ion Conductance Microscopy (SICM)	Uses a nanopipette; ion current changes with distance to surface and local ion concentration	10 nm –100 nm	Broad concentration range
				Primarily for topography, but concentration can be inferred in controlled conditions
	Ion-Selective Microelectrodes (ISMs)	Glass micropipette with ion-selective liquid membrane (e.g., for Ca^2+^, K^+^, Na^+^, Cl⁻)	100 nm –1 µm	µM to mM (e.g., Ca^2+^: 10^–7^ M to 10^–3^ M)
				Direct, quantitative, but can be slow
Local Electric Field	Vibrational Stark Effect (VSE) Reporters	A nitrile (C≡N) or carbonyl (C = O) group's vibrational frequency shifts with local electric field	~ 1 nm (molecular scale)	0.1 – 10 cm⁻^1^/(MV/cm)
				Provides atomic-scale field strength and direction via Raman/IR spectroscopy
	Nano-/Microelectrodes	A simple conductive electrode (Pt, carbon) used to measure local potential	10 nm –10 µm	mV to V
				Direct electrical measurement, but can be invasive and measures potential, not field directly
	Electrostatic Force Microscopy (EFM)	AFM tip measures electrostatic force	10 nm –100 nm	mV to V surface potential Maps potential/charge distribution on surfaces in air/vacuum

This table summarizes the typical operating ranges and characteristics of several key micro-environment probe techniques. These techniques complement each other and their application prospects in studying issues such as local pH changes in the hydrogen evolution reaction, ion concentration gradients on the surface of battery electrodes, or the electric field distribution within the electric double layer

*In situ* sensors provide high sensitivity and real-time monitoring, enabling the detection of trace substances and immediate observation of electrochemical changes. These techniques offer detailed information about the chemical environment and product structure, aiding in understanding reaction mechanisms and optimizing battery performance. However, they may be affected by the complex and dynamic electrochemical environment, causing measurement interference and noise. Other substances in the sample matrix can also interfere with sensing signals, affecting the accuracy and reliability. Moreover, the development and application of *in situ* sensing techniques require specialized equipment and expertise, limiting their accessibility and applicability in some research settings.

### Combination of Various *in situ* Techniques for Comprehensive Mechanistic Insights

Each *in situ* characterization technique offers unique capabilities. Electron microscopy (e.g., TEM, SEM, and STEM) provides high-resolution imaging of material morphology and structural evolution, while spectroscopy (e.g., XAS, Raman, and IR) reveals chemical environment and intermediate species dynamics at electrode interfaces. MS distinguishes species via mass-to-charge ratios, enabling precise product elucidation. A comparative analysis of these techniques is summarized in Table 3. However, conventional single-technique characterization provides only single-dimensional information, which is insufficient for comprehensive mechanism understanding. The challenging issue can be addressed by the combination of different *in situ* techniques, such as EC-AFM-Raman, GC/LC–MS, and AFM-STM, etc., which refer to the simultaneous or sequential use of multiple techniques during the actual occurrence of electrochemical reactions. This approach enables the acquisition of complementary data from different dimensions (space, time, information), facilitating a comprehensive understanding of reaction mechanisms. In electrochemical energy conversion (e.g., water electrolysis and fuel cells) and storage (e.g., lithium batteries) technologies, understanding performance metrics (such as capacity, cycle life, and catalytic activity) requires insight into the dynamic chemical/structural evolution of electrode materials, intermediates, and/or products*. In situ* combination of multimodal techniques provides unprecedented insights into our understanding of complex reaction mechanisms and device failure mechanisms.

**Table 3:** Features of various advanced *in situ* characterization techniques

	Technique	Advantages	Limitations	Refinements
Materials	TEM	High-resolution imaging	Sample preparation is complex and requires thin samples	FIB
		Ability to observe nanoscale structures	Vacuum conditions	ETEM, Liquid-cell TEM
		Multiple imaging modes	Expensive equipment	
	SEM	High-resolution imaging	Lower resolution compared to TEM	FIB-SEM
		Suitable for surface morphology analysis	Limited to surface analysis	ESEM
			Vacuum conditions	
		Simple sample preparation		
	STEM	Atomic resolution	Stringent sample requirements	FIB
		Multimodal imaging		Cryo-STEM
		Suitable for thick samples	Beam damage	AC-STEM
			Limitations of aberrations	
	LSCM	High spatial resolution	Limited penetration depth	LSFM
		Non-destructive testing	Photobleaching and phototoxicity	2PE/MPM
	AFM	Enables atomic-level imaging	Slow imaging speed	HS-AFM
			Limited to the imaging range	OM-AFM
		Provide abundant physical property information		
	STM	Atomic-level resolution imaging	High requirements for surface flatness	AFM
			Only conductive	
		Can image electronic states	samples can be observed	
	SECM	Provide information on chemical activity Work in a liquid—phase environment	Spatial resolution is limited	SECM- AFM Functionalized probes
			Limited to electroactive substances	
	APT	Atomic-scale resolution	A needle-shaped sample is required	FIB-SEM
			Electrically conductive sample	LA-APT
		Provide quantitative information		
	XRD	Non-Destructive	Not applicable to amorphous samples	PDF
		Rapid phase identification		
			Spatial resolution is limited	μ-XRD
	XPS	Surface sensitive	Detection depth is extremely shallow	iXPS, SA-XPS, NanoESCA
		Provide abundant chemical state information		
			Detection limits for certain elements	SIMS-XPS
				APXPS
			Vacuum conditions	
	REXS	Element specificity	Limited direct real-space imaging capability	CDI
		Extremely high sensitivity to weak superlattice signals		Ultrafast time-resolved REXS
			Conventional measurements are static	
	EIS	High sensitivity and non-destructive testing	Heavily relies on the equivalent circuit model	DRT analysis
		Provide quantitative kinetic parameters	Lack of spatial resolution	LEIS
	XAS	Provide abundant electronic and geometric structure information	Rely on synchrotron radiation source Complex equipment and data interpretation	DLSR
		Local structure probe		
Intermediates	IR	Used for chemical bond and functional group analysis	Relatively low spatial resolution	AFM-IR
				Thin-layer cell
			Sensitive to water	
		Suitable for polymers and organic compounds		
	Raman	Provides molecular vibrational information	Fluorescent background may interfere	SERS, TGRS
		Non-destructive testing		
			Weak Raman signal intensity	
				SERS, TERS
	EPR	High specificity and selectivity	The sample must have unpaired electrons	Spin labeling, Spin trapping
		Provide abundant structural, dynamic and environmental information	Relatively low sensitivity	
Products	NMR	Provides molecular structure information	Inherently low sensitivity	FT-NMR
		Suitable for both liquid and solid samples		
		Non-destructive		
	LC	High-resolution separation	Insufficient qualitative ability	LC–MS, HPLC
		Suitable for complex mixture analysis	Consume organic solvents, environmental unfriendliness	
	GC	High sensitivity and specificity	Not suitable for non-volatile or thermally unstable compounds	Derivatization
		High quantitative accuracy and repeatability		GC–MS
			Weak qualitative ability	
	MS	Excellent qualitative ability	Requires vacuum systems	
		Extremely high sensitivity and extremely low detection limit	Unable to distinguish isomers	
		Coupled with a variety of separation techniques		
Surroundings	Sensor	Real-time monitoring Customizable design Wide application range	Potential issues with stability and accuracy Some sensors have limited selectivity	MIPs

This table summarizes the advantages, limitations, and refinement strategies for various *in situ* characterization techniques, with improvement items presented in abbreviated form. The key to abbreviations and specific terms is provided below

FIB(Focused Ion Beam); ETEM(Environmental Transmission Electron Microscopy); AC-STEM(Spherical Aberration Corrected Scanning Transmission Electron Microscope); LSFM(Light-Sheet Microscopy); 2PE(2-Photon Excitation Microscopy); MPM(Multiphoton Microscopy); HS-AFM(High-Speed Atomic Force Microscope); LA-APT(Laser Pulsed Atom Probe Tomography); PDF(Pair Distribution Function); μ-XRD(Micro-X-ray Diffraction); SA-XPS(Small-Area X-ray Photoelectron Spectroscopy); iXPS(Imaging XPS); NanoESCA(Nano-Electron Spectroscopy for Chemical Analysis); SIMS-XPS(Secondary Ion Mass Spectrometry-XPS); APXPS(Ambient Pressure X-ray Photoelectron Spectroscopy); CDI(Coherent X-ray Diffraction Imaging); DRT(Distributed Relaxation Time); LEIS(Localized Electrochemical Impedance Spectroscopy); DLSR(Diffraction-Limited Storage Ring); TGRS(Time-Gated Raman); HPLC(High Performance Liquid Chromatography); MIPs(Molecularly Imprinted Polymers)

### AI-assisted* in situ* Characterization Techniques

While *in situ* characterization techniques enable observation under operating conditions, they often encounter challenges such as limited data quality, complex data interpretation, and slow analytical throughput. AI-assisted *in situ* characterization techniques represent a paradigm shift in scientific research, transforming these methods from mere “tools” into active “research partners.” This evolution is driven by the deep integration of AI’s computational power and cognitive reasoning with the real-time observational capabilities of *in situ* experiments [57, 58]. The enabling of *in situ* characterization techniques by artificial intelligence can be mainly elaborated from the following three aspects: 1) Feature extraction and identification: Machine learning (ML) identifies complex spectral features or structural details, such as defects and phase boundaries in imaging or spectral data. In techniques like *in situ* electron microscopy and NMR, where signals are weak and noise predominates, trained AI models effectively distinguish meaningful signals from background interference, thereby recovering clear and reliable data. In the above multimodal cases, AI algorithms (such as convolutional neural networks and recurrent neural networks) can effectively integrate multidimensional data streams from different techniques, often collected asynchronously. For example, through timestamp alignment and feature extraction, an AI model can integrate molecular vibration information from SERS and synchronously collected electrochemical current signals to achieve automatic classification and prediction of reaction mechanism pathways. 2) Accelerated data acquisition: Conventional high-resolution NMR requires prolonged signal-averaging times. AI models now enable intelligent reconstruction of high-quality spectra from sparsely sampled data, reducing acquisition time from hours to minutes. This breakthrough facilitates real-time monitoring of fast chemical processes. 3) Modeling and automated discovery: AI, integrated with computational methods, rapidly establishes structure–property relationships and accelerates catalyst screening. Furthermore, such models can be embedded in an autonomous experimental platform to dynamically adjust experimental parameters such as potential and temperature based on real-time multimodal feedback, so as to actively search for optimal reaction conditions or verify specific scientific hypotheses. AI-driven automation replaces labor-intensive experimentation, enhancing throughput while minimizing human bias, leading to more standardized and reproducible research workflows. This has significantly accelerated the process from “data collection” to “knowledge generation”, propelling fields such as materials science, chemistry, and physics into a new era of intelligent and automated discoveries . To assist readers in quickly understanding the characterization techniques discussed in the following application examples,  Table 4 summarizes the main abbreviations and full names of the *in situ* characterization techniques used throughout this work.

**Table 4:** The list of abbreviations for *in situ* characterization techniques

Abbreviations	Full words
TEM	Transmission Electron Microscope
SEM	Scanning Electron Microscope
STEM	Scanning Transmission Electron Microscopy
LSCM	Laser Scanning Confocal Microscopy
AFM	Atomic Force Microscope
STM	Scanning Tunneling Microscope
SECM	Scanning Electrochemical Microscopy
APT	Atomic Probe Tomography
XPS	X-ray Photoelectron Spectroscopy
XRD	X-ray Diffraction
REXS	Resonant Elastic X-ray Scattering
XAS	X-ray Absorption Spectroscopy
IR	Infrared Spectroscopy
EIS	Electrochemical Impedance Spectroscopy
EPR	Electron Paramagnetic Resonance
NMR	Nuclear Magnetic Resonance
LC	Liquid Chromatography
GC	Gas Chromatography
MS	Mass Spectrometry
SICM	Scanning Ion Conductance Microscopy
ISMs	Ion-Selective Microelectrodes
VSE	Vibrational Stark Effect
EFM	Electrostatic Force Microscopy
ETEM	Environmental Transmission Electron Microscopy
EDS	Energy Dispersive Spectrometer
FIB	Focused Ion Beam
AC-STEM	Spherical Aberration Corrected Scanning Transmission Electron Microscope
LSFM	Light-Sheet Microscopy
2PE	2-Photon Excitation Microscopy
MPM	Multiphoton Microscopy
HS-AFM	High-Speed Atomic Force Microscope
LA-APT	Laser Pulsed Atom Probe Tomography
PDF	Pair Distribution Function
μ-XRD	Micro-X-ray Diffraction
SA-XPS	Small-Area X-ray Photoelectron Spectroscopy
iXPS	Imaging XPS
NanoESCA	Nano-Electron Spectroscopy for Chemical Analysis
SIMS-XPS	Secondary Ion Mass Spectrometry-XPS
APXPS	Ambient Pressure X-ray Photoelectron Spectroscopy
CDI	Coherent X-ray Diffraction Imaging
DRT	Distributed Relaxation Time
LEIS	Localized Electrochemical Impedance Spectroscopy
DLSR	Diffraction-Limited Storage Ring
TGRS	Time-Gated Raman
HPLC	High Performance Liquid Chromatography
MIPs	Molecularly Imprinted Polymers
SERS	Surface-Enhanced Raman Spectroscopy
TERS	Tip-Enhanced Raman Spectroscopy
SHINERS	Shell-Isolated Nanoparticle-Enhanced Raman spectroscopy
ATR-SEIRAS	Attenuated Total Reflection Surface-Enhanced Infrared Absorption Spectroscopy
FT-IR	Fourier Transform Infrared Spectroscopy

This table systematically lists the main abbreviations for *in situ* characterization techniques used in the text along with their full names, greatly facilitating readers’ reference

## *In situ* Studies of EECSTs

### Fuel Cells

Fuel cells are devices that directly convert chemical energy into electrical energy and have the advantages of high efficiency and environmental friendliness. *In situ* characterization techniques can observe the electrochemical reaction processes on the surface of fuel cell electrodes in real-time, revealing the interaction mechanism between catalysts and reactants, and providing strong support for optimizing catalysts and improving fuel cell performance. Notably, the research examples covered in this review include both *in situ* and *operando* methods. To better distinguish between the two, we provide the following explanations. *In situ* Characterization: Refers to the real-time monitoring of materials or processes under a controlled (electro)chemical environment or stimulus. The test conditions may be simplified compared to those of actual devices. *Operando* Characterization: Specifically refers to the simultaneous performance measurement and real-time characterization under device-relevant operating conditions.

#### Oxygen Reduction Reaction (ORR)

**Fig. 5:**
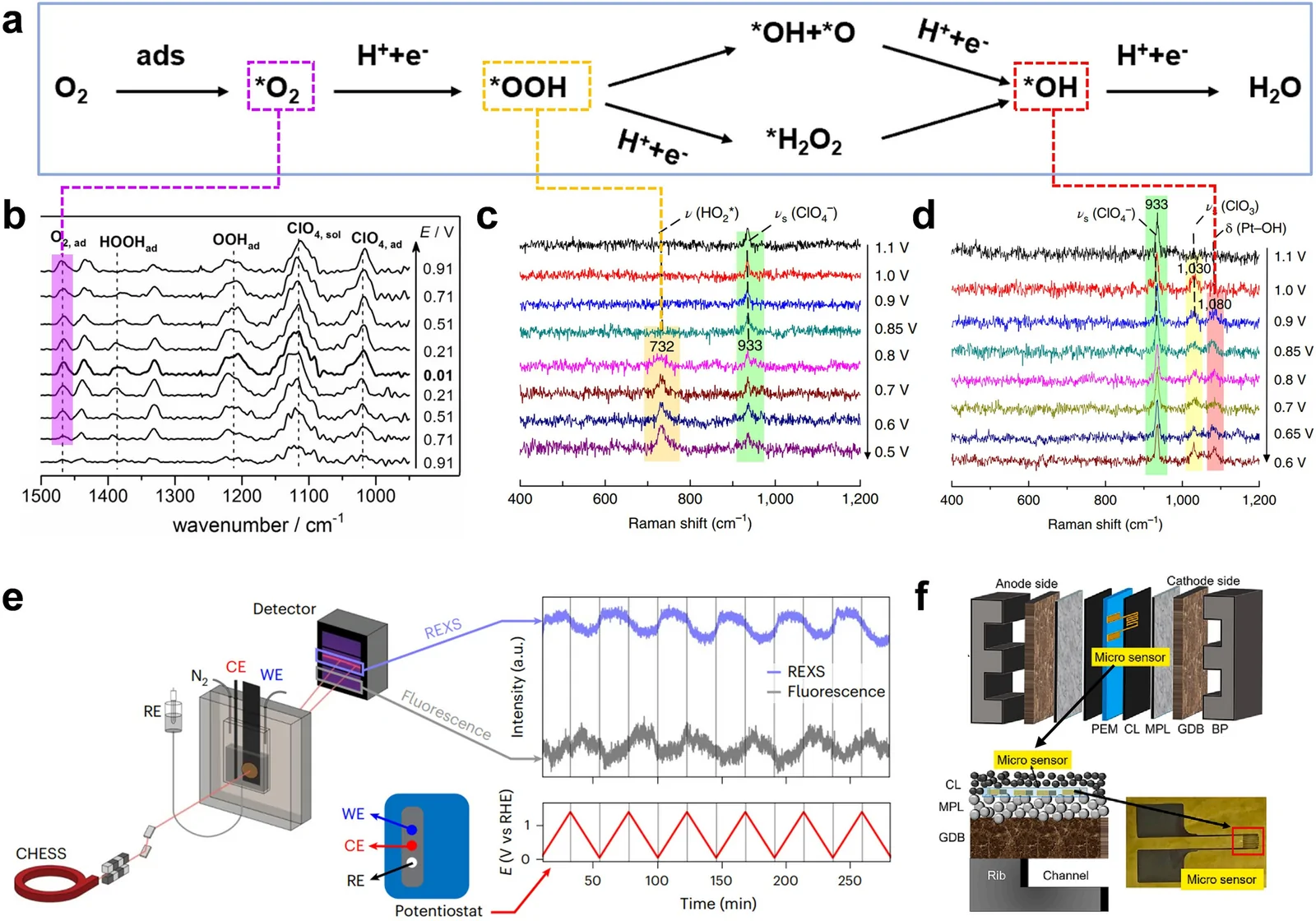
*In situ/operando* studies of the ORR processes by various characterization techniques. **a** ORR mechanism in an acidic medium. **b** ATR-IR spectra were recorded during the constant potential steps. Reproduced with permission from Ref. [67]. Copyright 2018, Wiley–VCH Verlag GmbH & Co. KGaA, Weinheim. EC-SHINERS spectra of the ORR system at **c** Pt(111) and **d** Pt(100) surfaces in a 0.1 M HClO_4_ solution saturated with O_2_. Reproduced with permission from Ref. [68]. Copyright 2018, Springer Nature. **e** *In situ* XRD and REXS. Reproduced with permission from Ref. [76]. Copyright 2020, Elsevier B.V. **f** Fuel cell configuration and micro-sensor position. Reproduced with permission from Ref. [78]. Copyright 2023, Elsevier B.V

Hydrogen-fueled proton exchange membrane fuel cells (PEMFCs), one of the most promising hydrogen energy conversion systems, can directly convert chemical energy stored in H_2_ into electrical energy with zero emissions, high conversion efficiency, and moderate operating temperature [59]. Unfortunately, the unfavorable kinetics of the cathode ORR severely hindered the practical applications of PEMFCs [60]. Compared to non-noble metal catalysts, Pt-based catalysts are the top choice for commercial applications because of their high-efficiency activity and long-term stability in acidic electrolytes [61]. Generally, there are two different ORR mechanisms based on the final products: the four-electron pathway to generate H_2_O, and a two-electron pathway to generate H_2_O_2_ [62]. The four-electron pathway can be further divided into the association mechanism and dissociation mechanism [63]. Taking acidic electrolytes as an example, the specific reaction steps of the four-electron and two-electron pathways often occur simultaneously, involving various intermediate species such as superoxide (O_2_^−^), peroxide (O_2_^2−^, OOH), and hydroxyl (OH) species [64]. The formation of these intermediates critically influences the performance and reaction mechanisms of ORR catalysts (Fig. 5a) [65, 66]. However, direct experimental evidence has long been lacking. Therefore, capturing key intermediate species through *in situ* techniques is essential to reveal the actual ORR pathways, providing valuable insights for improving catalyst performance.

The first step of ORR is the adsorption of O_2_ on the catalyst surface (Fig. 5a). Nayak et al. reported the direct evidence of adsorbed *O_2_ by *in situ* IR spectroscopy. The peak at 1468 cm^−1^ in Fig. 5b corresponds to the O–O stretching vibration of adsorbed O_2_, and the peaks at 1212 and 1386 cm^−1^ are assigned to the O–O stretching vibration of *OOH and OOH bending vibration of *HOOH, respectively [67]. After adsorbing on the active site, *O_2_ converts to *OOH via a proton-coupled electron transfer (PCET) step. Meanwhile, Dong et al. provided direct evidence for this viewpoint through *in situ* monitoring of the ORR processes on the Pt(*hkl*) surface via *in situ* SHINERS [68]. Only *OOH can be observed on Pt(111) surface (732 cm^−1^, Fig. 5c) which suggests a fast conversion of *OH to H_2_O and leads to superior ORR activity, while *OH without *OOH can be observed on Pt(100) (1080 cm^−1^, Fig. 5d) and Pt(110) surfaces which suggests that accumulation of *OH blocks the active sites and leads to an inferior activity. With the help of *in situ* SERS, Li’s group also directly observed *OOH on the Pt-based catalysts’ surface (Au@PtNi NPs, Au@Pd@Pt NPs, Au@PtCoSn NPs, etc.) and established the structure–activity relationship via the frequency-shift of *OOH [69–71]. Then, the adsorbed *OOH can be decomposed into *OH and *O (*O further converts to *OH) via a chemical process, or converted to HOOH via the PCET processes (O–O further dissociates to form two *OH) [72]. It follows that *OH is also a crucial intermediate species closely related to ORR performance. These results provide ideas for the rational design of efficient catalysts, that is, exposing more active surfaces.

In addition to activity, stability is another important parameter for evaluating the efficiency of catalysts. The catalyst failure, including the degradation, oxidation, and poisoning processes, is inevitable during the ORR processes, which results in the decay of the performance [73]. Clarifying the specific failure mechanism is significant for designing catalysts with high activity and long-term durability. Rizza’s group provided direct visual micrographs for the degradation processes of Pt nanoparticles during cyclic voltammetry curves (CVs) via *in situ* TEM [74]. As the number of CVs increases, the processes of the detachment of the Pt NP from a substrate, the coalescence of small Pt NPs to bigger ones, and the appearance of a new nanoparticle, respectively, are visible. Ran and co-workers reported a Fe_1_V_1_-NC catalyst with Fe-V atomic pair which can lead to side-on adsorption of O_2_ and directly dissociate the O = O to form *O, preventing the generation of reaction oxygen species (ROS), which suppresses the associated catalyst corrosions and enhances the stability. This was supported by the results of *in situ* attenuated total reflection surface-enhanced infrared absorption spectroscopy (ATR-SEIRAS) and *in situ* EPR, which showed no signal of *OOH and *OH can be observed [75]. Singer’s group explored the interaction between structure and oxidation state of Co-Mn spinel oxide electrocatalysts in ORR through multimodal *in situ* synchrotron radiation XRD and REXS (Fig. 5e) [76]. *In situ* XRD results demonstrated the reversible tensile strain induced during the dynamic reaction process, suggesting the structural robustness. *In situ* REXS results show that Co-Mn spinel oxide undergoes a dynamic confinement phase transition from cubic phase to tetragonal phase under applied voltage, which is related to the decrease in oxidation states of Co and Mn (serving as active sites for ORR). As for poisoning, SO_2_ is a common air impurity that is easily adsorbed on the catalyst surface and causes severe attenuation of ORR activity. Baturina et al. reported the electrochemical CVs of SO_2_ adsorption on Pt/VC (VC: Vulcan carbon) [77]. The charge of the H region gradually decreases as the number of CVs increases, indicating the blocking of Pt active sites. The *in situ* XANES of S was collected at different potentials. Only S^0^ is identified at 0.1 V while a mixture of S^0^ and SO_2_ is suggested at 0.5 V. When the potential increases to 0.7 V, an oxidation of adsorbed sulfur species ((bi)sulfate ions) can be detected, and only (bi)sulfate ions exist when the potential higher than 0.9 V. It indicates that oxidation at high potential is beneficial for removing the adsorbed SO_2_, thereby restoring catalytic performance.

As an important component of PEMFC, ORR exhibits strong irreversibility, which leads to the largest proportion of released heat of the cathode catalyst layer (CL) in the whole PEMFC [78]. Excessive temperature and variation during dynamic operation seriously impair the performance and durability of catalysts due to the unintended destructive state, such as ion dissolution, carbon corrosion, crack formation, and so on, making the large-scale application of fuel cells still challenging [79, 80]. Initially, commercial thermocouples (CTs) inserted into flow fields measured temperature variations over time [81]. However, CTs exhibited significant temperature lag relative to current variations, and their bulky size caused poor layer contact and gas leakage, making them unsuitable for catalyst-layer measurements [82]. To minimize interference, thin microsensors (5–50 μm), including film thermocouples and resistance thermal detectors, were developed for direct catalyst-layer temperature monitoring [83, 84]. Kim et al. demonstrated a flexible resistance temperature detector that enabled in-plane temperature distribution mapping without compromising fuel cell performance [85]. Ming et al. engineered ultrathin (~ 15 μm) resistance thermal detectors positioned at the catalyst/microporous layer interface (Fig. 5f) [78]. These sensors captured real-time temperature fluctuations during dynamic current changes, revealing a 1.6 °C rise from 200 to 1000 mA cm^−2^ and an additional 3 °C increase up to 1800 mA cm^−2^, with temperature recovery upon current reduction. Temperature variations were strongly influenced by cathode humidity and stoichiometric ratios but minimally affected by anode conditions, reflecting the greater mass transfer resistance and reactant sensitivity of cathodic ORR.

#### Hydrogen Oxidation Reaction (HOR)

**Fig. 6:**
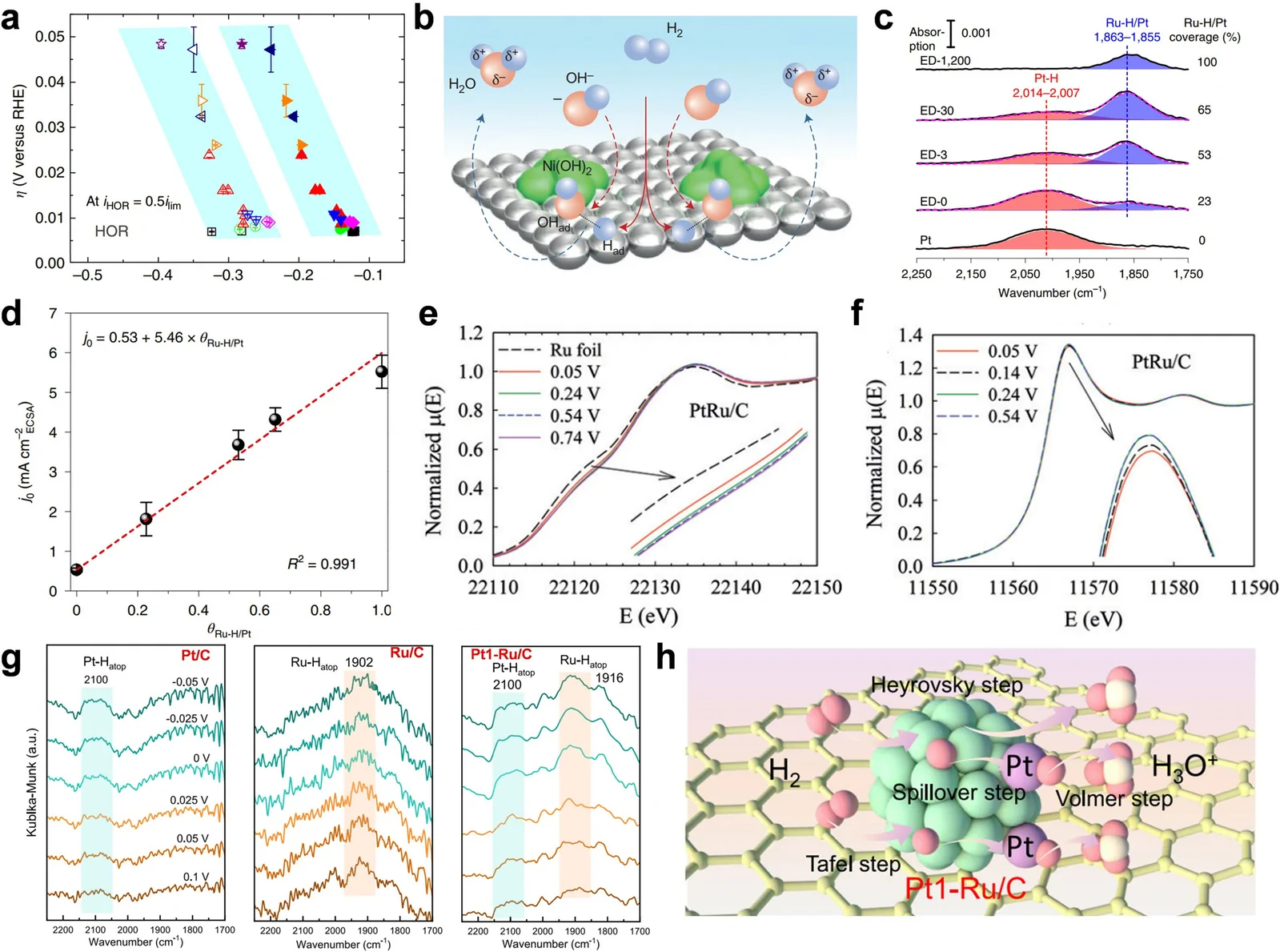
*In situ/operando* studies of the HOR processes by various characterization techniques. **a** The overpotential for the HOR in all pH-buffered electrolytes on Pt(110) (solid symbols) and (100) (open symbols) surfaces. Reproduced with permission from Ref. [89]. Copyright 2015, Springer Nature. **b** Schematic representation of the HOR on Ni(OH)_2_/Pt(111). Reproduced with permission from Ref. [90]. Copyright 2013, Springer Nature. **c** Fitting results of spectra recorded at 0 V on various electrode surfaces. **d** Linear fitting result between the calculated Ru–H/Pt coverage and specific *j*_*0*_. Reproduced with permission from Ref. [91]. Copyright 2021, Springer Nature. Ru K-edge XANES spectra **e** and Pt L3-edge XANES spectra of **f** Pt_1_Ru_1_/C collected in H_2_-saturated 0.1 M KOH at various potentials. Reproduced with permission from Ref. [92]. Copyright 2017, Wiley–VCH Verlag GmbH & Co. KGaA, Weinheim. **g** *In situ* synchrotron ATR-FTIR spectra of a Pt/C, Ru/C, and Pt1-Ru/C catalysts. **h** Schematic illustration of the tandem HOR processes on Pt1-Ru/C. Reproduced with permission from Ref. [93]. Copyright 2025, Springer Nature

Alkaline exchange membrane fuel cells (AEMFCs) have gained prominence owing to the high activity and stability of low-/non-platinum catalysts in alkaline environments [86]. There are two reaction pathways of alkaline HOR which are composed of three elementary reactions (H_2_ + 2 M* → 2 M-H_ad_; Heyrovsky step: H_2_ + OH^−^ + M* → M*-H_ad_ + H_2_O + e^−^; Volmer step: M*-H_ad_ + OH^−^ → M* + H_2_O + e^−^, M* represents the active site and H_ad_ represents the adsorbed H) [87]. The main difference between the two pathways is whether electrons participate in the first-step reaction. In the Tafel-Volmer pathway, H_2_ dissociates into 2H_ad_ without involving electrons, while H_2_ is converted to H_ad_ and H_2_O by losing an electron in the Heyrovsky-Volmer pathway. Early studies attributed HOR activity to hydrogen binding energy (HBE), where weaker Pt–H-bonding correlated with higher activity, as demonstrated by Sheng et al. through pH-dependent HBE trends (Fig. 6a) [88, 89]. However, HBE alone fails to explain alkaline HOR behavior. Markovic’s work on PtRu alloys and Ni(OH)_2_-modified Pt revealed enhanced activity, proposing a bifunctional mechanism where oxophilic sites (Ru, Ni(OH)_2_) adsorb *OH to accelerate *H removal (Fig. 6b) [90]. Despite progress, the dominant HOR mechanism remains debated due to insufficient direct evidence of OH/H intermediates, highlighting the need for *in situ* characterization to resolve these uncertainties.

Inspired by introducing oxophilic metals to improve HOR activity, Shao’s group synthesized a Ru-modified Pt electrode and monitored the electrochemical interface via *in situ* ATR-SEIRAS [91]. The result in Fig. 6c shows that Ru atoms not only play a role in tuning the electronic structure of Pt or oxophilic metal but also serve as active sites for H adsorption. The linear relationship between Ru–H/Pt coverage and exchange current density (*j*_*0*_) shown in Fig. 6d further confirmed this viewpoint. If Ru atoms only serve as the adsorption sites for *OH as proposed by the bifunctional mechanism, the relationship between Ru–H/Pt coverage and *j*_*0*_ should be volcanic rather than linear. Jia’s group observed the obvious up-shift of Ru K-edge (Fig. 6e) and down-shift of Pt L3-edge (Fig. 6f) via *in situ* XANES [92]. It is attributed that the adsorption of *OH on Ru sites promoted the desorption of *H from Pt sites (facilitating the oxidation of *H), which provides direct spectroscopy evidence for a bifunctional mechanism for HOR. Sun’s group designed a highly efficient tandem catalyst with Ru nanoclusters decorated with Pt single atoms (Pt1-Ru/C) and confirmed the crucial role of hydrogen spillover during the HOR process using *in situ* ATR-FTIR [93]. As shown in Fig. 6g, h, the peaks at around 2100 and 1900 cm^−1^ are assigned to the Pt-H_atop_ and Ru-H_atop_, respectively. The Pt-H_atop_ on the Pt/C surface decreased and even disappeared with the potential increasing (from –0.05 to 0.1 V) due to the hydrogen oxidation and desorption. The Ru-H_atop_ on Ru/C displays negligible changes, which can be attributed to the strong adsorption of *H on Ru. However, both peaks on Pt1-Ru/C weakened with the increased potential, suggesting that the hydrogen desorption on Ru was facilitated, which is closely related to the hydrogen spillover from Ru to Pt sites. Wang et al. constructed a PtNi alloy on Au nanoparticles and employed *in situ* SERS to monitor the HOR processes. The OH species were observed on the PtNi surface but not detected on the Pt surface, directly confirming that the doped oxophilic Ni promotes the adsorption of *OH to enhance HOR performance [94]. Lin’s *in situ* SERS study on Au@PtRu captured dynamic *OH intermediates (712–724 cm^−1^) and Ru valence shifts (+ 3 to + 4) during HOR, with activity tunable via Pt/Ru ratios [95].

**Fig. 7:**
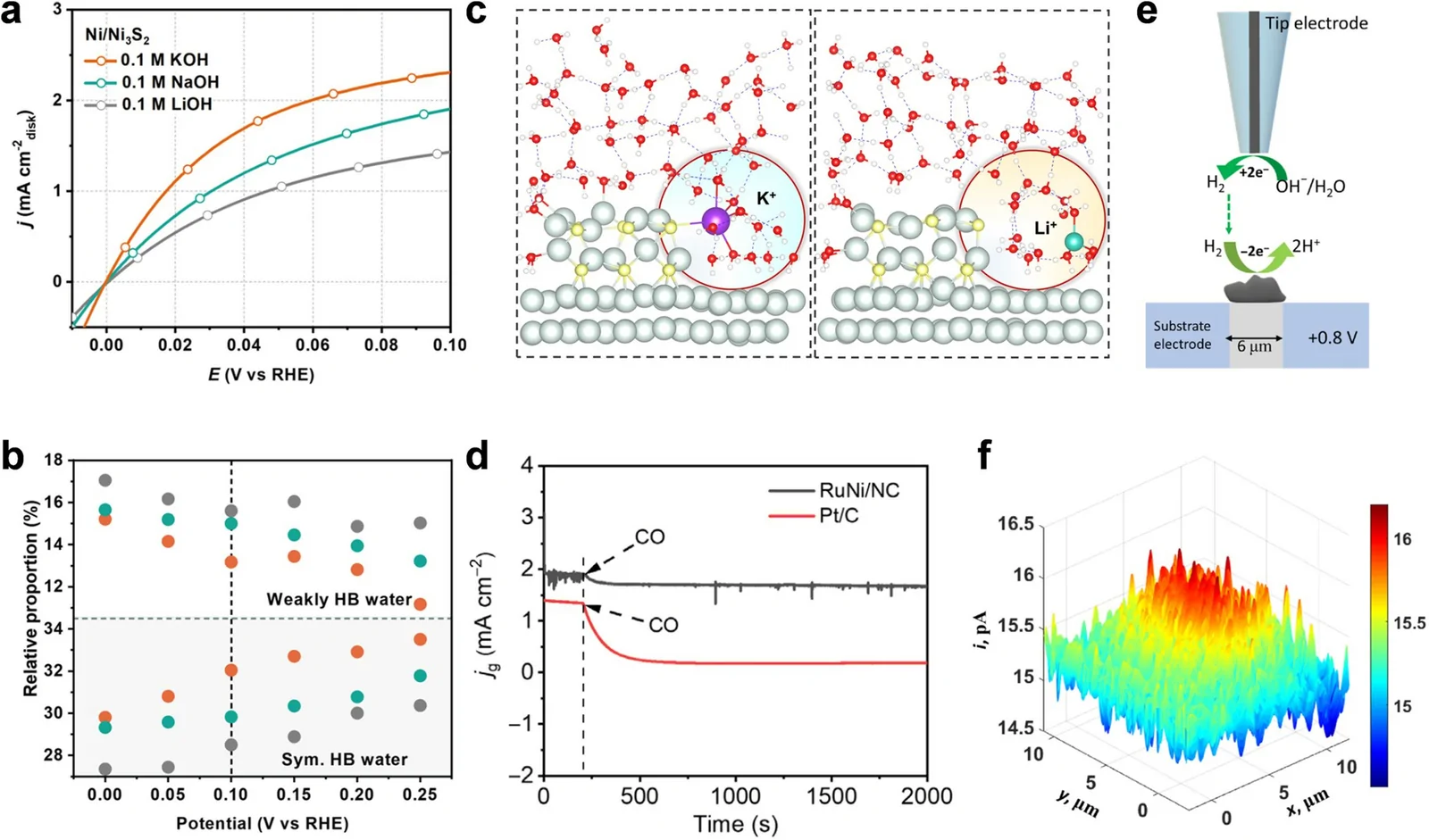
*In situ/operando* studies of the HOR processes by various characterization techniques. **a** Cation-dependent HOR polarization curves on the Ni_3_S_2_/Ni RDE electrode in H_2_-saturated aqueous electrolytes of 0.1 M KOH, NaOH, and LiOH. **b** Potential dependence of the relative fractions of weakly H-bonded water and symmetric H-bonded water. **c** Side view of the interfacial structures on Ni_3_S_2_/Ni in which K^+^ and Li^+^ ions are inserted, respectively. Reproduced with permission from Ref. [99]. Copyright 2025, American Chemical Society. **d** Current density-time chronoamperometry response of RuNi/NC and Pt/C in H_2_/200 ppm CO-saturated 0.1 M KOH solution at 50 mV. **e** Schematic representation of probing electrocatalytic activity for HOR of the RuNi/NC catalyst by the TG-SC mode of SECM experiments. **f** SECM TG-SC mode image of the RuNi/NC sample. The solution contains 0.1 M PB (pH = 10). *E*_S_ =  + 0.8 V, *E*_T_ =  − 1.2 V versus Ag/AgCl. Reproduced with permission from Ref. [87]. Copyright 2022, The American Association for the Advancement of Science

In addtition to *H/*OH intermediate species, the interfacial water and H-bonding structures at the electrode/electrolyte interface also play an important role in alkaline HOR [96]. Luo’s group prepared a face-centered-cubic phase Ru-based catalyst (*fcc*-RuCrW) and revealed that the adsorption strength of *OH is regulated by introducing oxyphilic Cr and W into Ru via *in situ* SEIRAS. The enhanced adsorption of *OH allows more free water molecules to be released into the adjacent gap region, which can improve the water connectivity and hydrogen-bond networks in the electric double layer (EDL), resulting in a superior HOR performance [97]. Yang et al. reported a RuS_2-x_ catalyst modified with S vacancies and revealed that the S atom in RuS_2-x_ is the actual active site for HOR through *in situ* Raman characterization (S–H bond was detected but Ru–H bond was not, indicating that H atoms were adsorbed on the S site during the HOR process [98]. The mechanism of H_2_O molecules at the electrode/electrolyte interface on HOR were further revealed through *in situ* SEIRAS. Men and co-workers designed a Ni_3_S_2_-island-encapsulated Ni catalyst (Ni_3_S_2_/Ni), and ab initio molecular dynamics simulations was employed to clarify that the Ni_3_S_2_/Ni with low surface charge density reduced the aggregation of cations which leads to a nonrigid and cation-uncrowded H-bonded network and results in an enhanced proton transfer kinetics and excellent HOR performance [99]. It was supported by their *in situ* SEIRAS results. Furthermore, the effect of different cations on HOR activity was studied; the activity trend is K^+^  > Na^+^  > Li^+^ (Fig. 7a). As shown in Fig. 7b, the proportion of strongly H-bonded water in KOH is higher, which is more conducive to proton transfer and facilitates PCET steps. On the contrary, the proportion of strongly H-bonded water in LiOH is higher, which destroys the connectivity of the hydrogen-bond network and results in lower HOR activity. This is consistent with the Ab initio molecular dynamics (AIMD) simulation results about the interface structures with cations (Fig. 7c).

Not only can the adsorption strength of intermediates affect the HOR activity, but it also has an impact on its stability and CO poisoning resistance. Han et al. identified Ru-Ni diatomic sites as optimal HOR active centers via DFT [87]. Their designed RuNi/NC catalyst demonstrated exceptional HOR activity, durability, and CO tolerance (Fig. 7d). *In situ* SECM (Fig. 7e) revealed H₂ oxidation at RuNi/NC substrates (Fig. 7f), with current surpassing background levels absent in NC controls, confirming Ru-Ni as active sites. *In situ* XAS attributed this synergy to enhanced H₂ dissociation and reinforced *OH adsorption. Guo’s group constructed an unconventional hexagonal close-packed-phase intermetallic Pd-based multi-metalline with homogeneously dispersed isolated Ru-O_3_ atomic sites, which were verified by EXAFS [100]. Combined with *in situ* SERS, it was found that the hcp intermetallic strengthens hydroxyl binding energy (OHBE) and water binding energy (H_2_OBE) while the Ru-O_3_ is profitable to weaken the intermediate adsorption energy. Modulated surface-adsorbed species adsorption leads to excellent robustness and CO tolerance. Wang’s group developed atomic Pt-functioned Ru nanoparticles and illustrated the anti-deactivation role of Pt via *operando* XAS and FT-IR [101]. The pure Ru nanoparticles are easily oxidized, which results in deactivation. The introduction of Pt improves the oxidation resistance of the nanoparticle surface, ensuring a water network channel for H oxidative release and consequently leading to a stable HOR performance.

### Water Electrolysis

Hydrogen production by water electrolysis is an important way to achieve sustainable energy utilization. *In situ* characterization techniques can deeply study the electrochemical reaction kinetics, intermediate product formation, and evolution on the catalyst surface during water electrolysis, which helps optimize hydrogen production technology by water electrolysis and improve hydrogen production efficiency.

#### Hydrogen Evolution Reaction (HER)

HER is an efficient and environmentally friendly method to provide a hydrogen source for achieving the hydrogen cycle [102]. However, compared with acidic electrolytes whose adsorbed hydrogen (*H) directly originates from the hydronium ions (H_3_O^+^), the *H in alkaline electrolytes needs to undergo the water dissociation step (Volmer process), which brings a higher energy barrier and leads to sluggish kinetics [103, 104]. Ruthenium (Ru)-based catalysts have received widespread attention due to their low price and excellent HER performance. For example, You et al. developed Ru nanoparticles grown *in situ* on carbon cloth (Ru-G/CC) via a one-pot solvothermal method, achieving a low overpotential of 40 mV at 10 mA cm^−2^, surpassing commercial 20 wt% Pt/C [105]. While its mixed amorphous-crystalline structure correlates with enhanced performance, the underlying mechanism remains unclear. Clarifying this requires probing structural evolution and interfacial water dissociation at the electrode-solution interface, yet direct observation of interfacial water dynamics is hindered by environmental complexity and bulk water interference.

**Fig. 8:**
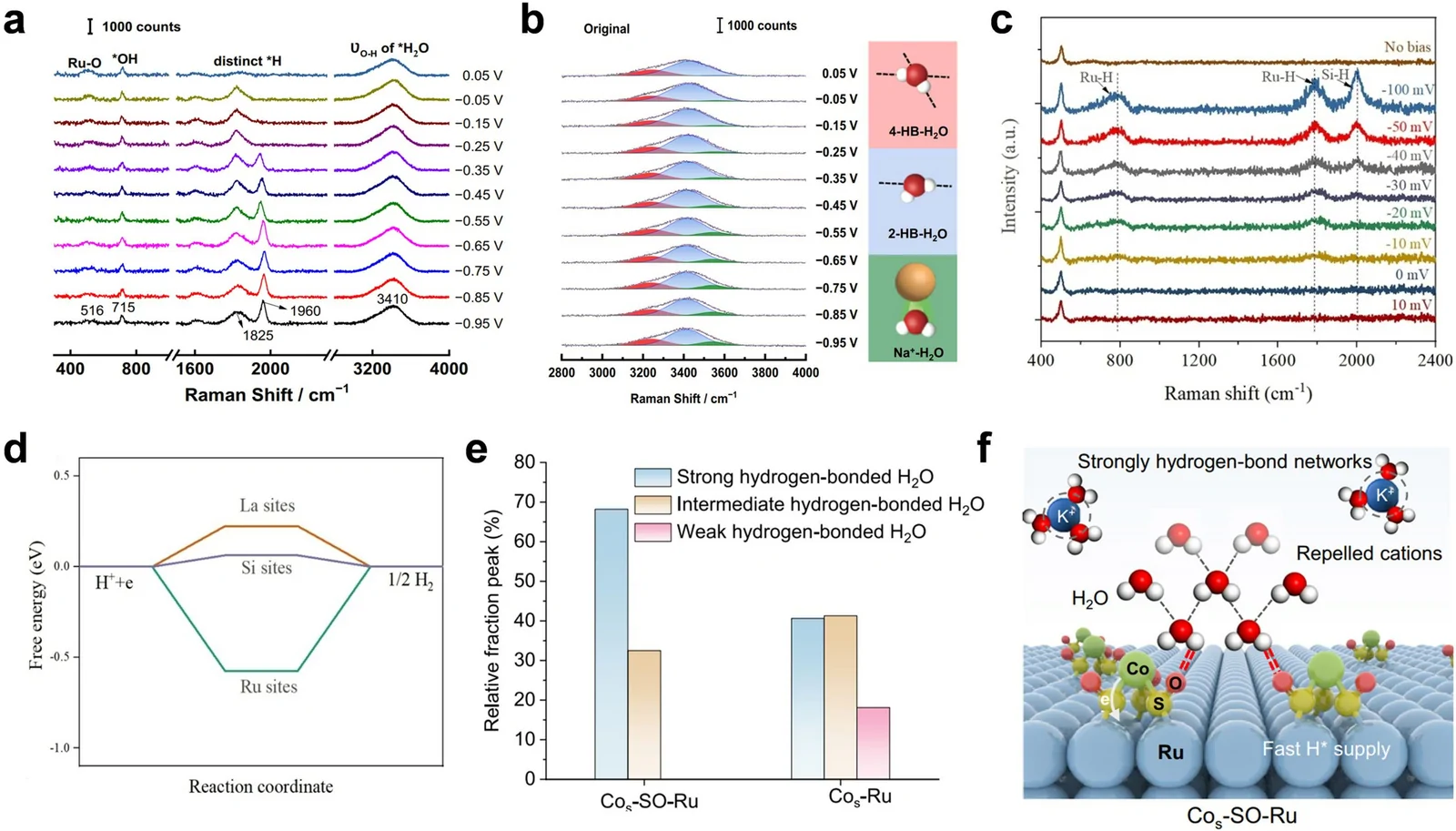
*In situ/operando* studies of the HER processes by various characterization techniques. **a** Raman spectra of the alkaline HER processes at 55 nm Au@2.5 nm Ru electrode surfaces. **b** Raman spectra of interfacial water at original Ru surfaces, Gaussian fits of three O–H stretching modes (ν_O-H_) of 4-coordinated hydrogen-bonded water (4-HB·H_2_O), 2-coordinated hydrogen-bonded water (2-HB·H_2_O), and hydrated Na^+^ ion water (Na·H_2_O) are shown in red, blue, and green, respectively. Reproduced with permission from Ref. [107]. Copyright 2023, Springer Nature. **c** Raman spectra of the LaRuSi catalyst at various potentials (vs. RHE) under HER conditions in 1.0 M KOH. **d** H adsorption-free energy at different sites of LaRuSi. Reproduced with permission from Ref. [110]. Copyright 2017, Wiley–VCH GmbH. **e** The relative fraction of different H_2_O configurations. **f** Scheme of hydrophilic sulfo-oxygen bonds bridged Co-Ru atomic pairs for regulating interfacial hydrogen-bond network. The dotted lines represent hydrogen bonds. Reproduced with permission from Ref. [111]. Copyright 2023, Springer Nature

Wang et al.’s work on the Pd single crystal surface by *in situ* SHINERS revealed that interfacial water mainly consists of H-bonded and hydrated Na^+^ ion water. The dynamic variation from random to the ordered distribution of interfacial water structure, which is influenced by the bias potential and Na^+^ ion cooperation, was observed during the HER processes. The highly ordered interfacial water structure is instrumental in minimizing additional work, allowing maximized electrochemistry energy conversion and leading to an enhanced HER performance [106]. With the assistance of a “borrowing strategy”, Chen et al. utilized *in situ* SERS to probe the HER mechanism of Au@Ru nanoparticles, capturing dynamic spectral evidence of Ru valence states (Ru(0), Ru(n +)), *H and *OH intermediate species, and interfacial water with different structures (4-HB⋅H_2_O, 2-HB⋅H_2_O and Na⋅H_2_O) were obtained directly (Fig. 8a, b) [107]. They revealed the mechanism of the valence state tuning effect on interfacial water and intermediates with enhanced HER performance. Specifically, the large work function of Ru(n +) and the local cation tuning effect of Na^+^ ion water promote the water dissociation step. The more moderate adsorption energies of interfacial water, *H, and *OH on Ru(n +) surfaces also facilitate the HER activity.

Enhancing HER performance through Ru-based catalysts via doping, alloying, or hybridization has proven effective. Zhang et al. synthesized multilayered hcp-phase RuNi nanosheets via a one-pot solvothermal method, achieving a record-low overpotential of 15 mV at 10 mA cm^−2^, outperforming commercial Ru/C and Pt/C [108]. Liu et al. [109] engineered Ru₉₀Ni₁₀ nanoparticles on graphene oxide (Ru₉₀Ni₁₀/rGOPs), where Ni’s oxophilicity induced surface reconstruction into Ru-Ni(OH)₂ multisites, and compressive strain from Ni weakened H adsorption, achieving an ultralow overpotential of 6 mV at 10 mA cm^−2^. Zhong et al. identified the active sites of LaRuSi for HER using *in situ* Raman spectroscopy [110]. At potentials below 0 mV (e.g., − 10 and − 20 mV), peaks at 783 cm^−1^ (Ru–H bending) and 1791 cm^−1^ (Ru–H stretching) emerged (Fig. 8c). A Si–H stretching vibration (2004 cm^−1^) appeared at − 30 mV, with all peaks disappearing upon potential removal. Theoretical calculations revealed La shifts Ru’s d-band center upward, inducing a negative Ru valence that strengthens H adsorption. However, Si sites exhibited near-ideal H adsorption energy (ΔGₕ = 0.063 eV, Fig. 8d), corroborated by weaker Si–H Raman signals, ultimately confirming Si, not Ru, as the dominant active sites. Inspired by metalloproteins in natural enzymes, Zhang’s group constructed a cobalt ruthenium atomic pair through sulfo-oxygen bridging (Co_s_-SO-Ru), forming a 3D hydrophilic network on the Ru nanoclusters surface and successfully regulating the dynamic structure of water molecules at the electrode–electrolyte interface [111]. As the relative fraction of different H_2_O configurations analyzed by *in situ* ATR-SEIRAS results shown in Fig. 8e, there is a stronger hydrogen-bonded H_2_O on Co_s_-SO-Ru via the formation of -S = O⋅⋅⋅H_2_O than that on Co_s_-Ru, suggesting the strengthened hydrogen-bond network in EDL, which can speed up the proton supply and *OH diffusion at the electrode–electrolyte interface and enhance the reaction kinetics. Moreover, this strong hydrogen-bond network also relieves the accumulation of Ca^2+^ and Mg^2+^ to prevent the formation of Ca(OH)_2_ and Mg(OH)_2_ at the reaction interface (Fig. 8f), improving the stability of the electrode during seawater conditions.

**Fig. 9:**
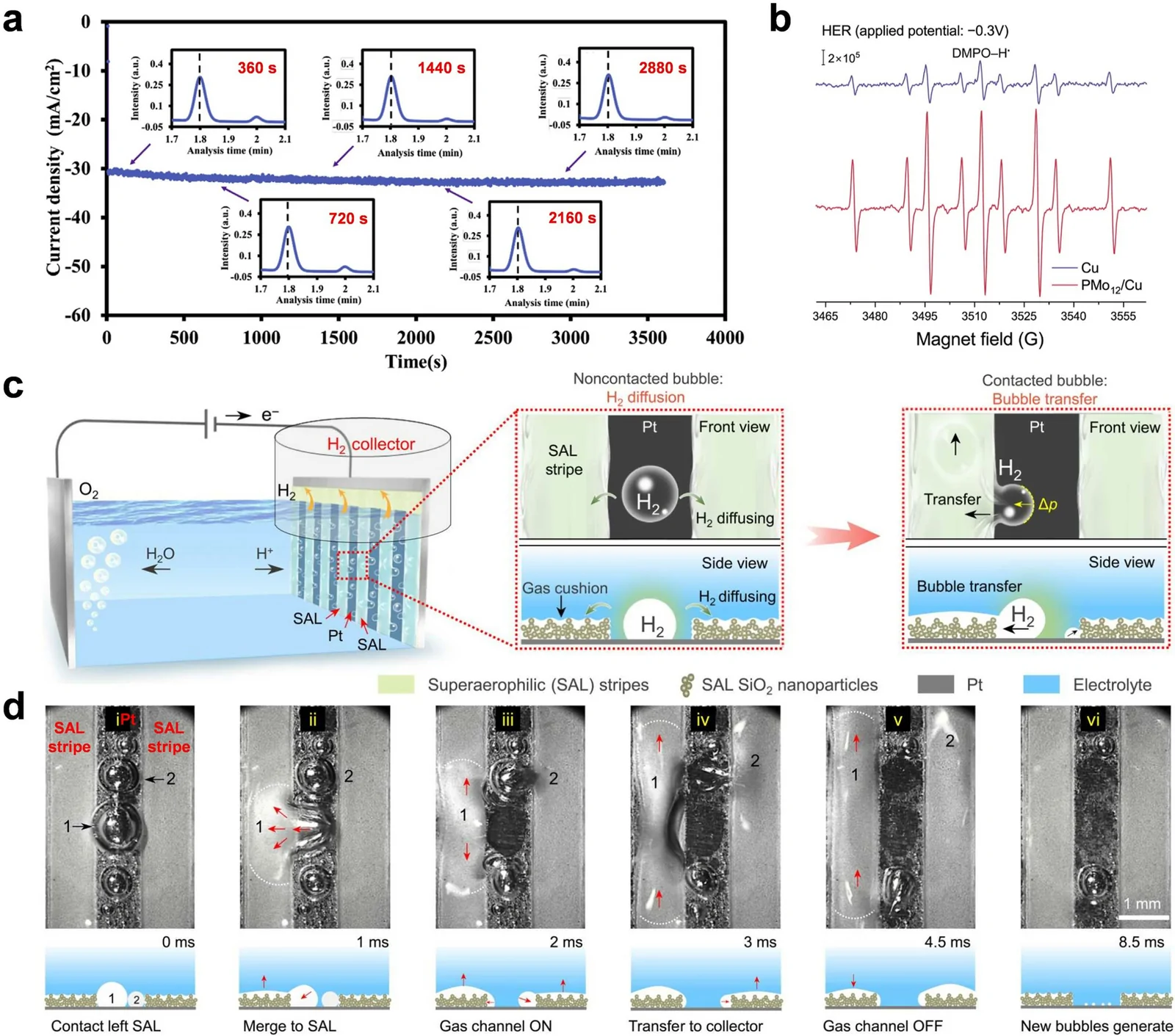
*In situ/operando* studies of the HER processes by various characterization techniques. **a** The chronoamperometry curve of GO@Ni electrode at -0.5 V vs. SCE with the *in situ* GC measurement of hydrogen gas evolution at 360, 720, 1440, 2160, and 2880 s. Reproduced with permission from Ref. [112]. Copyright 2019, Elsevier Ltd. **b** *Quasi-in situ* EPR trapping of HER on Cu and PMo_12_/Cu. Reproduced with permission from Ref. [113]. Copyright 2025, American Chemical Society. **c** Schematic of the SAL/flat Pt electrode with enhanced mass transfer. **d** *In situ* optical observation of bubble transfer (indicated by red arrows) on the SAL/flat Pt electrode. Reproduced with permission from Ref. [118]. Copyright 2023, American Association for the Advancement of Science, AAAS

Transition metal-based materials (oxides, sulfides, phosphides) are promising low-cost, high-efficiency catalysts for hydrogen evolution. Among these, Ni-based and Mo-based catalysts excel due to their affordability, conductivity, and catalytic activity. For instance, Sawangphruk et al. developed a 3D graphene oxide-coated nickel foam (GO@Ni), achieving an overpotential of 83.2 mV at 10 mA cm^−2^, with *in situ* GC confirming a hydrogen evolution rate of 10^−7^ mol cm^−2^ s^−1^ (Fig. 9a) [112]. Sun’s group designed a PMo_12_/Cu heterogeneous catalyst and utilized 5,5-dimethyl-1-pyrroline-N-oxide (DMPO) as a free radical scavenger; the free radical intermediates in the hydrogenation process of HER were studied by *quasi-in situ* EPR [113]. As shown in Fig. 9b, a much stronger DMPO-H* signal can be observed on PMo_12_/Cu than that on Cu during the HER process, indicating its superior *H generation ability. Luo et al. [114] reported a brand-new hydroxide-mediated NiMoFe electrocatalyst (h-NiMoFe) that takes place only 97 mV overpotential at 1000 mA cm^−2^. They employed *in situ* XAS, quasi-*in situ* XPS, and DFT to clarify the mechanism of enhanced HER performance, that is, the relative percentages of metallic Ni decrease in samples after HER and tend to form Ni(OH)_2_, which is beneficial for promoting the water dissociation step. This aligns with Ze et al.’s *in situ* SERS study on Au@Ni(OH)₂, where OH_ad_ participation and Ni(OH)_2_-to-NiO transformation under HER conditions confirmed Ni(OH)_2_’s role in water dissociation [115].

Optimizing the adsorption/desorption of intermediate species on the electrode/electrolyte interface to promote H_2_ generation is currently a widely studied direction. However, the serious adhesion of H_2_ bubbles on the electrode surface would lead to a “dead area” and cause the catalyst to “deactivate” [116, 117]. Therefore, it is crucial to quickly remove H_2_ bubbles from the reaction system and reduce the H_2_ concentration at the electrode surface to promote the HER process. Zhang and co-workers designed a superaerophilic/superaerophobic (SAL/SAB) cooperative electrode composed of SAL stripes and plat Pt (SAB electrocatalytic regions) [118]. As shown in Fig. 9c, the HER process takes place in the Pt region, and the generated H_2_ bubbles are transported to the H_2_ collector by SAL stripes. When formed bubbles are small (noncontacted bubbles), the diffusion can be facilitated due to the short diffusion distance, benefiting from the gas cushion layer at the SAL stripe. As the bubbles grow, driven by the asymmetric Laplace pressure between the bubble and the gas cushion, they will contact with the SAL stripes and transfer through them promptly, which is clearly observed in *in situ* optical results (Fig. 9d). Such rapid bubble diffusion facilitates the migration of H^+^ from electrolyte to electrode surface, resulting in outstanding HER performance.

#### Oxygen Evolution Reaction (OER)

Unlike the HER at the cathode, OER at the anode is a four-electron-proton transfer process with sluggish kinetics, which greatly limits the practical application of water electrolysis [119, 120]. Based on the results of previous studies, the OER mechanism over catalysts with different electronic structures can be divided into two types: conventional adsorbate evolution mechanism (AEM) and lattice-oxygen-mediated mechanism (LOM) [121]. The biggest distinction between them is that the coupling mode of O–O bond. The former follows the pathway of *OH, *O, *OOH, O_2_ and the O–O is coupled via the formation of *OOH whose O atoms both come from H_2_O while the latter follows the pathway of OH, O_2_^2−^, O_2_ and the O–O couples directly from an adsorbed O and a lattice O [122, 123]. *In situ* techniques such as Raman and IR provide an opportunity for identifying whether OER undergoes a pathway of AEM or LOM.

**Fig. 10:**
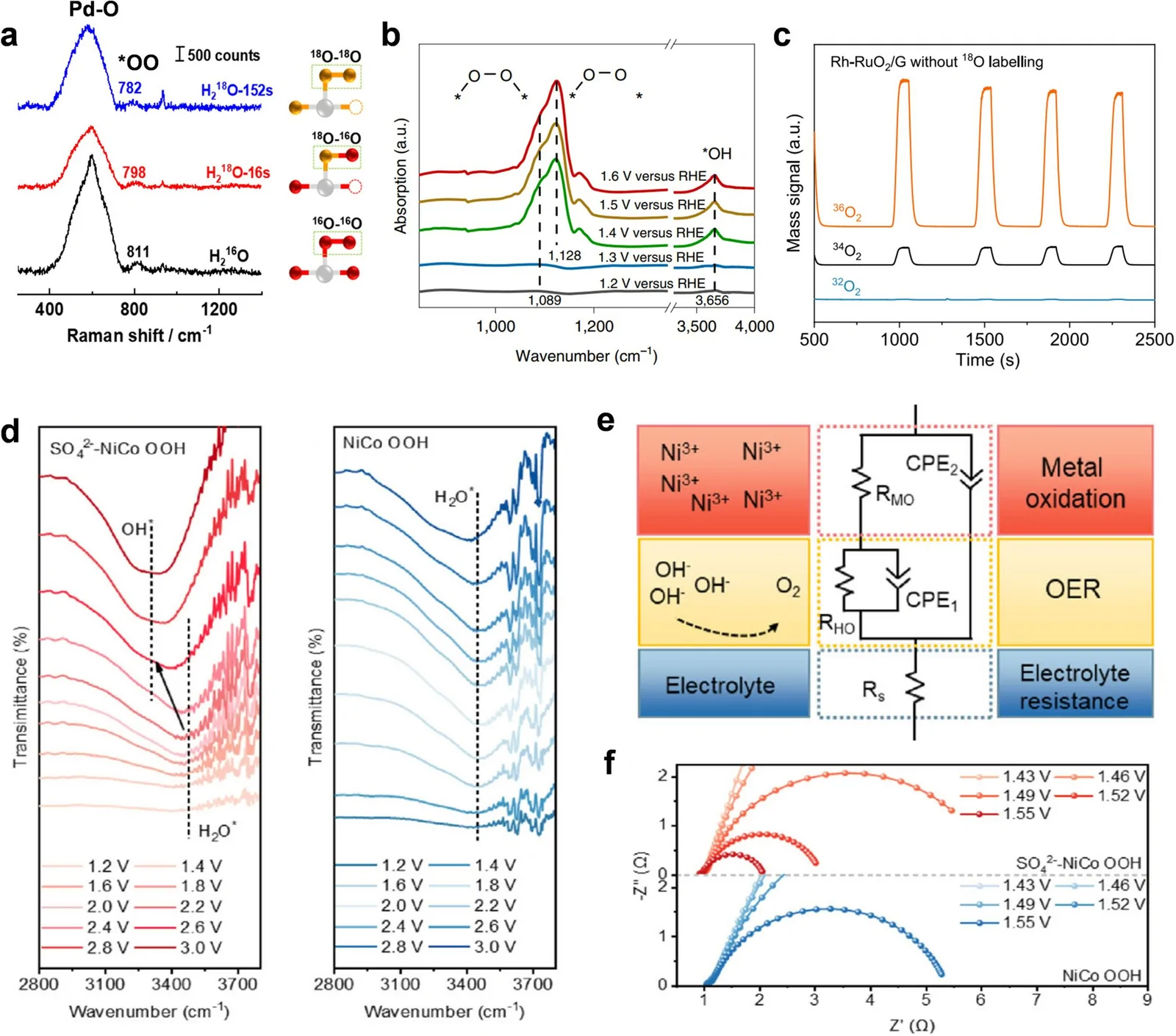
*In situ/operando* studies of the OER processes by various characterization techniques. **a** H_2_^18^O isotopic Raman spectra of 2 ML Pd in 0.1 M HClO_4_ (Ar-saturated, 1.4 V vs. Ag). H_2_^18^O-16 s and H_2_^18^O-152 s represent the spectra after replacing the electrolyte for 16 s and 152 s, respectively. Adsorption configuration of *OO on the Pd/Au surface on the right. Gray, red, and orange spheres represent the Pd, ^16^O, and ^18^O atoms, respectively. Reproduced with permission from Ref. [124]. Copyright 2024, Wiley–VCH GmbH. **b** *In situ* FT-IR spectra of Ru/MnO_2_. Reproduced with permission from Ref. [126]. Copyright 2021, Springer Nature. **c** DEMS signals of ^36^O_2_, ^34^O_2_, and ^32^O_2_ for Rh-RuO_2_/G in H_2_^18^O aqueous sulfuric acid electrolyte within four times of LSV at 1.1–1.9 V (vs. RHE). Reproduced with permission from Ref. [127]. Copyright 2023, Springer Nature. **d** *In situ* FT-IR spectra of SO_4_^2−^-NiCo OOH and NiCo OOH. **e** The equivalent electric circuit and **f** the fitted potential-dependent Nyquist plots of *R*_*MO*_ and *R*_*HO*_. Reproduced with permission from Ref. [134]. Copyright 2024, Wiley–VCH GmbH

Sun et al. monitored the electrooxidation processes on a Pd single crystal via *in situ* SERS. The results of *in situ* Raman confirmed the formation of surface oxides (PdO_x_) and the *OO peroxy intermediate in the deep oxidation region with higher potential [124]. Furthermore, the direct spectral evidence of ^16^O-^18^O obtained in an *in situ* ^18^O isotopic Raman experiment (Fig. 10a) elucidates that the oxygen atoms in PdO_x_ participate in the generation of surface-adsorbed peroxide species and initiate subsequent oxygen generation. Hu’s group employed a similar *in situ* isotopic Raman experiment [125]. The Co^16^OOH was first transferred to Co^18^O_2_ in ^18^O-KOH at 1.75 V, and then the electrolyte was replaced with ^16^O-KOH. With the applied potential increasing from 1.45 to 1.75 V, the Raman peak of CoO_2_ and O–O both shift to the higher frequency gradually, but are not completely the same as the frequency of the Co^16^O_2_ sample. They believed that the incomplete isotope exchange may be assigned to: not all sites on the catalyst surface are active; higher potential results in more active sites; the exchange process is slow on a time scale. Lin and co-workers obtained the information on O–O bond (1089 cm^−1^), metal-O–O (1128 cm^−1^), and OH (3656 cm^−1^) whose intensity increased dramatically with the potential increasing over 1.4 V via *in situ* FT-IR (Fig. 10b), suggesting that these are two key intermediates which are closely related to the OER processes [126]. Together with the theoretical calculations and other characterizations, they revealed that the OER undergoes the OPM pathway on the Ru/MnO_2_ surface, which involves direct O–O coupling, resulting in a reduced energy barrier. *Operando* DEMS measurements are another efficient method to verify whether lattice oxygen is involved in the OER processes. For example, Wang et al. conducted the ^18^O isotope labeling DEMS to investigate the OER processes on Rh-RuO_2_/G. As the results show in Fig. 10c, a main mass signal of ^36^O and a low signal of ^34^O without ^32^O were observed, revealing that the lattice oxygen atom is not involved in the formation of O_2_ during the OER processes [127]. Bao’s group employed multi-*in situ* techniques to dynamically track oxygen evolution on Ru_T_Ir_V_/CoOOH (Ru single atoms (SA) anchored on the three-fold facial center cubic (fcc) hollow sites and Ir SA anchored on the oxygen vacancy (V_o_) on CoOOH) [128]. *In situ* XANES spectroscopy showed that the absorption edge of Ru shifted positively and the coordination number increased, while the absorption edge position and coordination number of Ir single atoms did not change significantly during the reaction, indicating that Ru participated as an active site in the OER process, while Ir mainly played a stabilizing intermediate role rather than directly participating in the redox reaction. There is no significant change of CoOOH characteristic peaks in *in situ* Raman spectroscopy during the OER process, indicating that there is no phase transition or reconstruction. The direct evidence of *OOH key intermediates in *in situ* ATR-SEIRAS indicates the AEM. An obvious blue-shift of *OOH frequency compared with the Ru_T_/CoOOH catalyst, indicating that Ir single atoms weaken the adsorption strength of *OOH through hydrogen bonding and reduce the reaction energy barrier. Furthermore, *in situ* ^18^O isotope-labeled DEMS experiments showed that the main signals of all samples corresponded to ^32^O_2_ (^16^O-^16^O), indicating that the OER process followed the AEM. Furthermore, Lv’s group developed a strategy for integrating *in situ* multimodal characterizations and machine learning to clarify the reaction mechanism of Ru/TiMnO_x_ electrode [129]. Machine learning models extract multidimensional features from experimental measurements and structural characterization as inputs (such as element molar ratios, electrochemical features, crystallographic features and so on), output overpotentials and deactivation rates to achieve performance prediction, and establish structure-performance relationship. The structural evolution was detected via *in situ* Raman, and the A1g mode around 666 cm^−1^ was observed which demonstrates the formation of Ru–O-Mn. The *-O–O and *-O–O-* intermediates observed in the *in situ* ATR-FTIR, which is consistent well with the oxide path mechanism (OPM). The results of *in situ* ^18^O-labeling DEMS futher supports this OPM-related pathway in Ru/TiMnO_x_.

Besides intermediate species, understanding the evolution mechanisms of interfaces between electrode materials and electrolytes is also vital to establishing the structure-performance relationship [130]. Among these, anion modulation plays a vital role in positively persuading the reconstructed species’ OER performances [131]. For example, Feng et al. proposed a Fe_MOFs_-SO_3_ and attributed the enhancement of OER performance to the H^+^ capture by -SO_3_ from *OH or *OOH [132]. Xue et al. reported a sulfate-functionalized RuFeO_x_ (S-RuFeO_x_) and revealed that the sulfate dopants can weaken the adsorption energy of the *OO-H and stabilize lattice oxygen via *in situ* ATR-SEIRAS spectra [133]. Lin et al. designed an oxyanion-functionalized NiCo OOH catalyst and employed a series of *in situ* characterizations to investigate the OER process [134]. They elucidated that the introduced SO_4_^2−^ results in more free H_2_O enrichment on the catalyst surface and then engages in the OER process through *in situ* isotope-labeled Raman spectroscopy. *In situ* FT-IR was used to investigate the OH enrichment effect of the sulfate-induced solid–liquid interface. As shown in Fig. 10d, a peak at 3320 cm^−1^, which is assigned to the *OH species, can be observed on the SO_4_^2−^-NiCo OOH surface as potential increases, while it cannot be detected on the NiCo OOH surface, suggesting the formation and accumulation of *OH after introducing SO_4_^2−^ in the NiCo OOH surface. Furthermore, *in situ* Electrochemical impedance spectroscopy (EIS) was conducted to quantitatively evaluate the OH-transfer. As shown in Fig. 10e, f, the *R*_*HO*_ and *R*_*MO*_ are related to the hydroxyl oxidation and metal site oxidation processes, which are much lower in SO_4_^2−^-NiCo OOH than those in NiCo OOH, suggesting more OH-transfer. This multi-*in situ* technique proved that the functionalized oxygen anions on the catalyst surface can bond with H_2_O molecules in the electrolyte to form an H-bonded network, which bridges the gap at the solid–liquid interface and facilitates the OH-migration and enrichment, resulting in an outstanding OER performance.

**Fig. 11:**
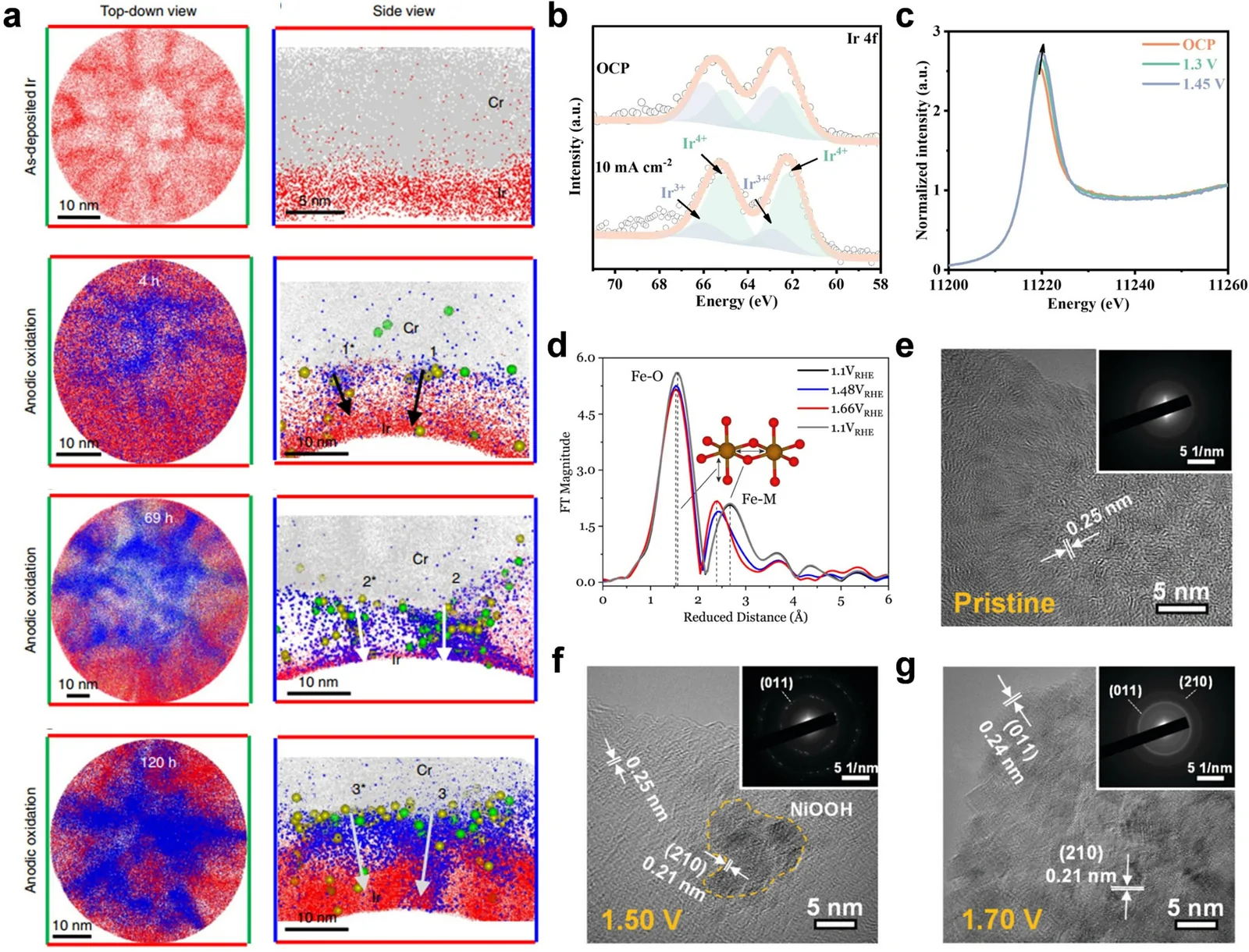
*In situ/operando* studies of the OER processes by various characterization techniques. **a** APT data of as-deposited Ir film and Ir oxides formed by anodic oxidation for 4 h, 69 h and 120 h. Reproduced with permission from Ref. [136]. Copyright 2023, Springer Nature. **b** Quasi-*in situ* Ir 4*f* XPS profiles for Ni-Ir DAC in OCP and post-tested at 10 mA cm^−2^ for 10 min. **c** *Operando* XANES spectra for Ni-Ir DAC recorded at OCP, 1.30 V, 1.45 V. Reproduced with permission from Ref. [138]. Copyright 2024, Wiley–VCH GmbH. **d** Fourier transformed EXAFS spectra of Fe K-edge for Ni_*x*_(Fe_1-*x*_)O_*y*_H_*z*_ were recorded *operando* at varied electrode biases. Reproduced with permission from Ref. [139]. Copyright 2025, American Chemical Society. **e–g** HRTEM images of Ce-NiBDC/OG before electrocatalysis and after applying different voltages. Inset: Corresponding SAED patterns. Reproduced with permission from Ref. [141]. Copyright 2020, Royal Society of Chemistry

The OER processes often involve catalyst surface reconstruction [135], a critical process for identifying active sites and elucidating reaction mechanisms. Recent advances in *in situ* techniques have enabled real-time monitoring of structural and compositional changes during OER. For example, Mayrhofer’s group has probed the structural transformation process of the first atomic layers from deposited Ir film to surface IrO_2_ layers at a near-atomic scale for OER via APT. As shown in Fig. 11a, the IrO_2_ tends to grow at grain boundaries and gradually transforms from oxide nanoparticles to a thicker oxide film with the anodic oxidation time increasing [136]. Zhang’s group employed *in situ* XAS and *in situ* ^57^Fe Mössbauer spectroscopy to monitor the OER processes on the surface of hierarchical FeVO_x_/NP [137], and the increase of the Fe oxidation state (the formation of Fe^4+^) was observed. Xu et al. investigated the surface chemical state evolution of Ni-Ir diatomic catalysts (DAC) during OER via quasi*-in situ* XPS [138]. The Ir 4*f* results as shown in Fig. 11b, the ratio of Ir^4+^/Ir^3+^ increased obviously upon OER. This is also supported by the results of the *operando* XANES spectra in Fig. 11c. The white line position shows a positive shift with the potential increasing, suggesting an increased Ir chemical state. Koroidov’s group performed *operando* XAS to detect the Fe-site state of Ni_*x*_(Fe_1-*x*_)O_*y*_H_*z*_ during the OER processes [139]. The results in Fig. 11d showed that the electronic and structural properties varied, but the alteration was reversible when the potential was cycled between 1.10 and 1.66 V (RHE). Zhu’s group reported the first real-time nanoscale observation of OER on the Mn_2_O_3_ surface by utilizing *in situ* liquid TEM [140]. They directly observed the generation and development of bubbles around the catalyst during the OER processes and the formation of a new surface layer on the catalyst whose thickness gradually increases over time, realizing the visualization of the structure and composition evolution during the OER processes. Cheng et al. also confirmed the phase transformation of catalysts by *in situ* microscopy [141]. The lattice fringe spacing of 0.25 nm in Fig. 11e is assigned to the (221) plane of NiBDC, but it became distorted with an unobvious diffraction ring when a 1.3 V potential was applied. Then, a new well-defined lattice fringe spacing of 0.21 nm appeared (assigned to the (210) plane of NiOOH) when the potential increased to 1.5 V with a polycrystalline ring, which corresponds to the (011) plane of NiOOH (Fig. 11f), illustrating the new phase formation. Furthermore, the whole nanosheet shows distinct lattice fringe spacing of NiOOH (0.21 and 0.24 nm correspond to the (210) and (011) planes, respectively, Fig. 11g). This phenomenon was also observed in the results of *in situ* Raman, that is, the peaks at 1615 and 1441 cm^−1^ (originate from Ce-NiBDC) disappeared with the peaks at 560 and 477 cm^−1^ (Ni–O bonds in NiOOH) appearing when the applied potential increased from 1.0 to 1.45 V.

### Carbon Dioxide Reduction (CO_2_RR)

The electricity-driven electrochemical conversion of CO_2_ not only provides a promising pathway for carbon neutrality to mitigate environmental pollution in the future but also a sustainable way to achieve value-added fuels and chemical feedstocks for energy storage and utilization on demand [142, 143]. As is well known, in contrast to mono-carbon products (CO, CH_4_, etc.), multi-carbon products, including hydrocarbons and oxygenated compounds with significant energy density (C_2_H_4_, C_2_H_5_OH, n-C_3_H_7_OH, etc.) are more valuable in solving energy crises [144]. The major bottleneck in the formation of multi-carbon compounds depends on the C–C coupling step during the CO_2_RR processes, which is closely related to the adsorption strength and coverage of *CO [145]. Thus, it is essential to develop the catalysts with optimal binding strength of *CO to speed up the coupling kinetics and effectively improve the selectivity toward multi-carbon products.

**Fig. 12:**
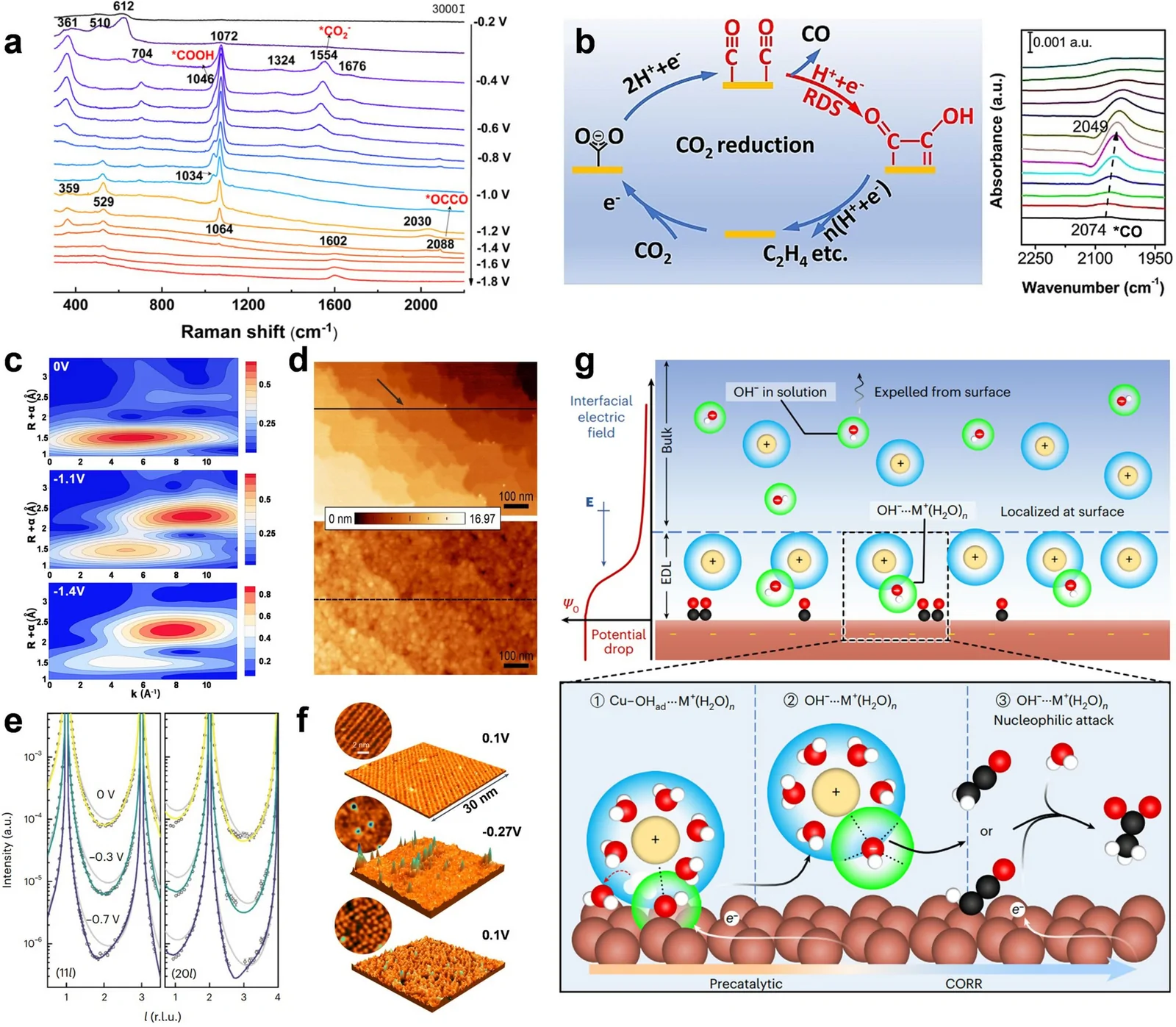
*In situ/operando* studies of the CO_2_RR processes by various characterization techniques. **a** Raman spectra of Cu(110). Reproduced with permission from Ref. [151]. Copyright 2022, Royal Society of Chemistry. **b** Schematic diagram of CO_2_ reduction procedure and ATR-SEIRAS spectra of Cu(OH)_2_-D. Reproduced with permission from Ref. [153]. Copyright 2020, Wiley–VCH GmbH. **c** Wavelet transforms for the *k*^*3*^-weighted Cu K-edge EXAFS signals at different potentials. Reproduced with permission from Ref. [157]. Copyright 2022, Springer Nature. **d** AFM images of electropolished Cu(100) recorded in air and after immersion in CO_2_-saturated 0.1 M KHCO_3_ at OCV. Reproduced with permission from Ref. [159]. Copyright 2020, Wiley–VCH Verlag GmbH & Co. KGaA, Weinheim. **e** SXRD measurements of the experimentally obtained CTRs (11* l*) and (20* l*) (circles) and the corresponding best fits (solid colored lines). **f** Three-dimensional and top-view (circular inset) STM images of the freshly prepared surface at different potentials. Reproduced with permission from Ref. [161]. Copyright 2023, Springer Nature. **g** Proposed mechanism for high acetate generation under alkaline CO_2_RR. Reproduced with permission from Ref. [163]. Copyright 2023, Springer Nature

Cupric-based materials are known to be the most promising CO_2_RR electrocatalysts, which can significantly improve the selectivity of conversion of CO_2_ into multi-carbon products due to the suitable binding energy of *CO [146–148]. Traditionally, CO_2_RR is thought to proceed via two sequential steps (CO_2_ → CO and CO → C_2+_) on the same site. Recently, Cui and co-workers investigated the adsorption behavior of CO_2_ molecules on the surface of different metal-doped graphitic C_3_N_4_ catalysts (27 kinds of metals including Cu) via machine learning assisted infrared/Raman spectroscopy technology, and revealed that stronger adsorption and charge transfer of the adsorbed CO_2_ is beneficial for performance improvement [149]. Xu’s group provided convincing experimental evidence via *in situ* SEIRAS and isotopic controlled characterization that the CO reduction reaction can be promoted by the presence of CO_2_ and there are at least two types of sites during CO_2_RR, that is, one site (Cu_CO2_) favors the CO_2_ → CO and the other site (Cu_CO_) is active in CO → C_2+_ [150]. Surface facets and structural effects play a critical role in the CO_2_RR. Zhao et al. reported that the CO_2_RR reaction pathway on Cu(*hkl*) is different via *in situ* SHINERS [151]. On Cu(110), Cu_2_O reduction (510 and 612 cm^−1^) precedes the appearance of *CH_2_CHO and *OCCO intermediates, indicating C_2+_ product formation (Fig. 12a). In contrast, only *COOH and *CO were detected on Cu(111), favoring C_1_ products. Meanwhile, Gao et al. designed oxide-derived Cu crystals with (100)/(111) interfaces, which exhibit enhanced *CO adsorption and lower C–C coupling barriers compared to individual facets, boosting C_2+_ selectivity [152]. Zhong et al. confirmed this facet effect using *in situ* ATR-SEIRAS [153]. A Cu(OH)_2_-derived catalyst with stepped Cu(110) and Cu(100) surfaces showed stronger linearly bonded CO (CO_L_) adsorption (higher wavenumber) than CuO- or Cu_2_O-derived catalysts (Fig. 12b), facilitating C–C coupling and C_2+_ production. Yang et al. investigated structural dynamics using *operando* electrochemical STEM (EC-STEM) on 7 nm Cu nanoparticles [154]. The high fraction of metallic Cu nanograins and abundant grain boundaries provided undercoordinated sites, resulting in sixfold higher C_2+_ selectivity compared to 18 nm nanoparticles. Zheng’s group detected the dynamic CO_2_RR processes on Cu catalyst solid–liquid interfaces in liquid-cell TEM. A fluctuating liquid-like amorphous interphase was observed directly, which resulted in the surface of the catalyst restructuring via interphase dynamics [155]. Lei et al. monitored the CO_2_RR processes via *operando* Raman and XRD on three Cu precursors (Cu(OH)_2_-, Cu_2_(OH)_2_CO_3_-, and CuO-derived Cu) [156]. The appearance of Cu(0) in Raman results and the fingerprint peaks of metallic Cu in XRD results when three samples were at their optimal reaction potential indicate that all three precursors are transformed into Cu(0) and no Cu oxide is involved during the CO_2_RR processes.

Beyond crystal facets and structural effects, electronic structure regulation is critical for enhancing CO_2_RR activity and selectivity. Su et al. synthesized CuO clusters on N-doped carbon nanosheets (Cu/N_0.4_C), achieving a 73% Faradaic efficiency (FE) for C_2+_ products [157]. N-doping induces charge-asymmetric sites, promoting *CH_3_ formation as a key intermediate for ethanol production, as confirmed by *in situ* FT-IR and DFT. *In situ* XAS and XPS revealed catalyst restructuring, with Cu_2_-CuN_3_ identified as the active site post-reduction. At − 1.1 V, a Cu-Cu bond peak (~ 2.4 Å) emerged, while Cu–N/O bonds diminished (Fig. 12c). XPS showed Cu(II) reduction to Cu(I)/Cu(0), with AES confirming Cu(I) dominance during CO_2_RR. Moreover, Qiao’s group reported a 2D-3D ensemble machine learning strategy combined quantum chemical calculation, artificial intelligence (AI) clustering and experiment for establishing the structure-performance relationship of Cu-based catalysts via the various complex intermediates during reaction process [158]. Based on rapidly and accurately built a big data set of over 45,000 data point (adsorption configurations and adsorption energy), it was revealed that the asymmetric coupling mechanism is superior to symmetric coupling on the Cu-based catalysts and the performance can be further improved through doping engineering. Importantly, it has been experimentally confirmed that the CuAgNb catalyst exhibits excellent C–C coupling performance which is predicted via big dataset analysis. It was found in most reported studies that the surface state of catalysts is not fixed and invariable but reconstructed during the reaction processes. Simon et al. provide *in situ* nanoscale potential-dependent structural transformations on Cu(100) surface via EC-AFM measurement [159]. The rough surface of Cu(100) by oxidation through immersing in the CO_2_-saturated bicarbonate solution at open-circuit potential (OCP) is visible in Fig. 12d. The same group further revealed that the Cu single crystals with atomically ordered surfaces are preferred to yield H_2_ while it favors the generation of hydrocarbons after inducing the defects, steps, and roughness [160]. Magnussen et al. Used *in situ* surface X-ray diffraction (SXRD) and STM to study Cu(100) reconstruction in CO_2_-saturated KHCO_3_ [161]. At − 0.3 V, crystal truncation rods (CTRs) shifted, indicating surface evolution (Fig. 12e). At − 0.7 V, asymmetric CTRs and increased roughness were observed, supported by STM images showing irreversible disorder upon returning to 0.1 V (Fig. 12f). The fresh Cu(100) surface exhibited an ordered layer of co-adsorbed carbonate anions and water. Upon applying a CO_2_RR potential (− 0.27 V), the surface became highly disordered. Returning to 0.1 V did not restore the original ordered layer, leaving a short-range ordered structure and demonstrating irreversible surface changes.

In addition to the intermediate species involved in the reaction, the interactions between the electrode surface and electrolyte are also crucial for shaping the microenvironments of electrochemical interfaces, directly influencing the reaction pathway and selectivity [162]. Focusing on the microenvironmental regulation mechanism of CO_2_RR on Cu catalyst, Yang’s group found that a non-covalent complex (OH_ad_⋅⋅⋅M^+^(H_2_O)_n_, M^+^ is the electrolyte cation) formed by surface hydroxyl (OH_ad_) and interfacial water via *in situ* Raman spectroscopy combined with isotope labeling experiments [163]. The complex is converted into OH^−^⋅⋅⋅M^+^(H_2_O)_n_ retained in the double layer at the catalytic potential, which significantly enhance the selectivity of acetate over other C_2_ products through nucleophilic attack on the key intermediate *HC = C = O (Fig. 12g). Xu’s group have quantitatively determined the impact of cations on the key thermodynamic and kinetics variables of CO adsorption (enthalpy and entropy) on Cu under electrochemical conditions with different alkali metal cations through *in situ* SEIRAS [164]. CO adsorption becomes increasingly unfavorable in the sequence Li^+^  > Na^+^  > K^+^  > Cs^+^ with the increase of enthalpy from Li^+^ to Cs^+^. Different metal cations affect the RDS by regulating the stability of the initial and transition states in opposite directions, thereby altering the overall CO_2_RR rate. Resasco’s group investigated the electrode/electrolyte interfaces of CO_2_RR in the non-aqueous electrolyte (organic alkylammonium cations) by the kinetic, spectroscopic and theoretical calculations [165]. It is revealed that the interfacial field strength can be changed by the cation-electrode distance, which affects the kinetically relevant CO_2_ activation step and the rate of CO formation. Wang et al. developed a local pH detection technique for the oxygen evolution reaction (OER) using CN–dye hybrid electrochemiluminescence (ECL) emitters [166]. The method offers high selectivity, sensitivity (with sub-second temporal resolution), and oxidation resistance. It enables real-time visualization of pH gradients near RuO_2_ electrocatalysts, reveals catalyst degradation mechanisms induced by proton accumulation, and introduces kₐₚₚ as a quantitative descriptor for the OER rate.

**Fig. 13:**
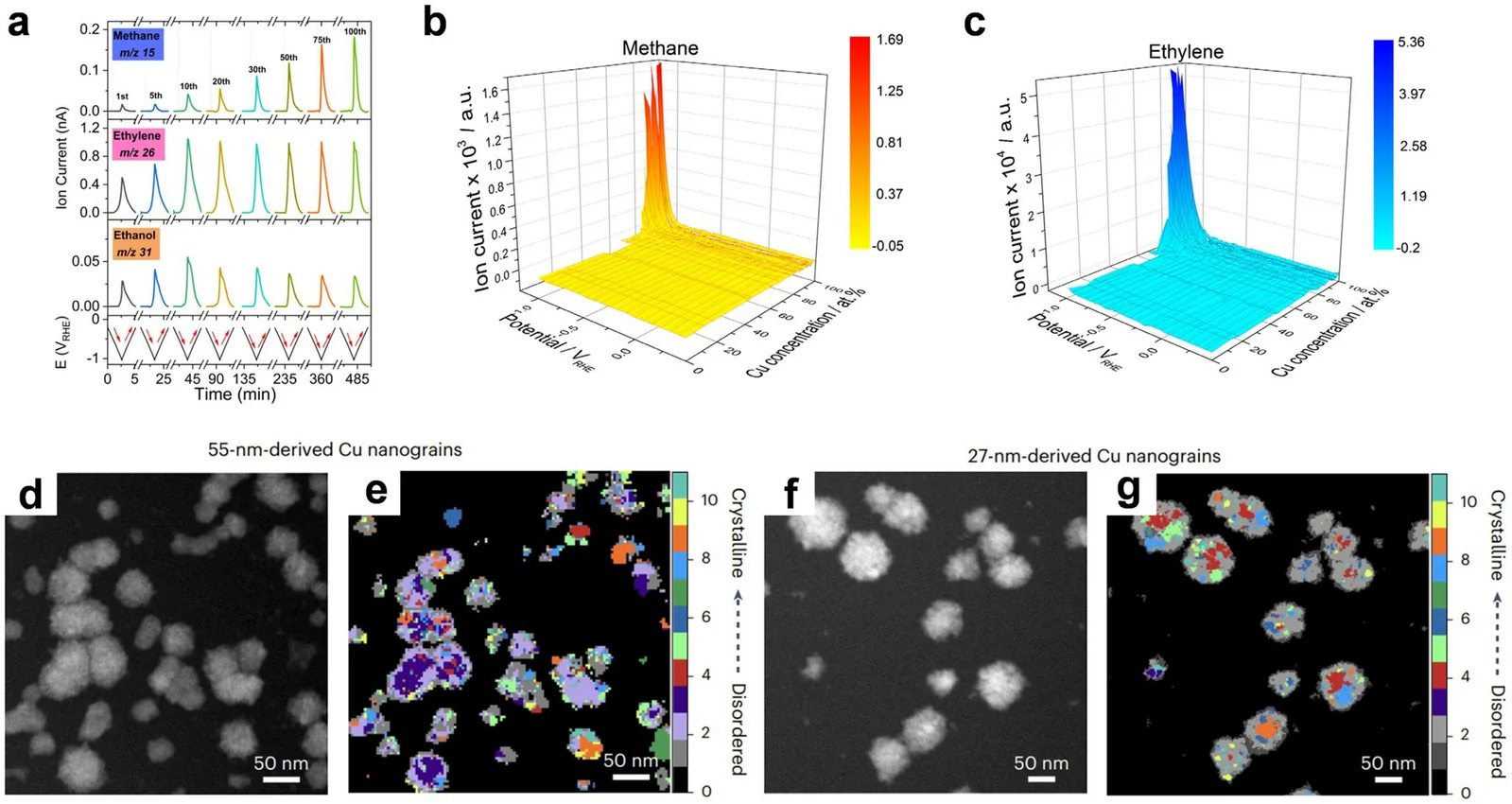
*In situ/operando* studies of the CO_2_RR processes by various characterization techniques. **a** *Operando* DEMS sweep data obtained during CO_2_RR on CuO NS catalysts. Reproduced with permission from Ref. [168]. Copyright 2021, Springer Nature. MS signals of **b** methane and **c** ethylene for the potential sweep experiments in dependence on potential and composition. Reproduced with permission from Ref. [169]. Copyright 2016, Elsevier Inc. (**d, f**) *Operando* HAADF-STEM images and (**e, g**) 4D-STEM clustering analysis of nanograins derived from (**d, e**) 55 nm and (**f, g**) 27 nm nanocubes at − 1 V. Reproduced with permission from Ref. [174]. Copyright 2021, Springer Nature

Furthermore, the product detection also plays a vital role in understanding the CO_2_RR dynamics. *In situ* MS is widely employed in monitoring the evolution of reaction products and the local surroundings of reaction interfaces in real-time [167]. For example, Strasser’s group revealed the onset potentials (where product generation sets in) of a variety of products by DEMS, which had not been reported before [168]. Figure 13a shows the relationship between the transient mass ion currents and the function of cycle number and time of CH_4_, C_2_H_4,_ and ethanol. The production of CH_4_ continues to increase, while the production of C_2_H_4_ and ethanol increased in the first hour cycles and peaked in the 10th cycle. A series of Cu-Co thin-film materials was prepared by Grote et al., and the effect of added Co in the Cu-Co thin-film material on selectivity was investigated [169]. The formation of various C_1_ and C_2_ products during the CO_2_RR processes was detected by an *in situ* online electrochemical mass spectrometer (OLEMS), and an interesting shift of selectivity was observed (Fig. 13b, c). The generation of ethane takes priority over that of methane at a low Co atom ratio (between 5 and 15%), resulting in a significantly increased proportion of C_2_ products. The generation of hydrogen gradually strengthens and dominates as the Co content further increases. Choi et al. detected C_3+_ products (allyl alcohol) during the CO_2_RR on a phosphorus-rich copper catalyst using *in situ* GC–MS, achieving a Faradaic efficiency of 66.9% [170]. *In situ* Raman spectroscopy further identified formaldehyde as a key intermediate formed at the copper oxide/hydroxide interface of the catalyst.

With the rise of artificial intelligence (AI), the combination of machine learning (ML) and *in situ* characterizations has received increasing attention which can avoid the limitations of the accumulation of errors from obtaining the information from spectral signals and the lack of a quantitative structure-performance relationship [171, 172]. Cuenya’s group employed the ML approach with *in situ* EXAFS data and constructed and trained an artificial neural network (NN) to realize the unambiguous distinction between fcc (Cu-rich alloy) and non-fcc (Zn-rich alloy) type structures data in CuZnO_x_ materials, which is difficult to distinguish by using the *in situ* XAS technique alone, as similar elements in the periodic table have similar photoelectronic properties [173]. Combined with the *in situ* SERS results, the presence of Zn was found to be beneficial for the stabilization of cationic Cu(I) species, and the formed Cu(0)/Cu(I) interfaces contribute to efficient electrocatalytic CO_2_ conversion to complex multi-carbon products. Yang’s group employed *operando* EC-STEM to study the dynamic structural evolution of 55 and 27 nm Cu cubes during the CO_2_RR process and illustrated that both kinds of nanocubes were completely reconstructed into metal Cu nanoparticles [174]. With the assistance of ML, the complexity of polycrystalline active sites was explored using *operando* electrochemical four-dimensional (4D) STEM technology. It was revealed that most of the nanoparticles derived from 55 nm Cu cubes were mainly composed of polycrystalline copper with different crystallinity (Fig. 13d, e), while a considerable portion of the copper nanoparticles derived from 27 nm Cu cubes were almost amorphous/disordered (Fig. 13f, g). Moreover, the *CO and its changes during the CO_2_RR process can be directly observed in *in situ* SERS, revealing the effect of *CO in the migration of Cu and the formation of Cu clusters. Such a combination of multi-model *operando* methods and AI techniques paves the way for understanding complex interfacial mechanism under reaction conditions.

### Lithium-ion Batteries

**Fig. 14:**
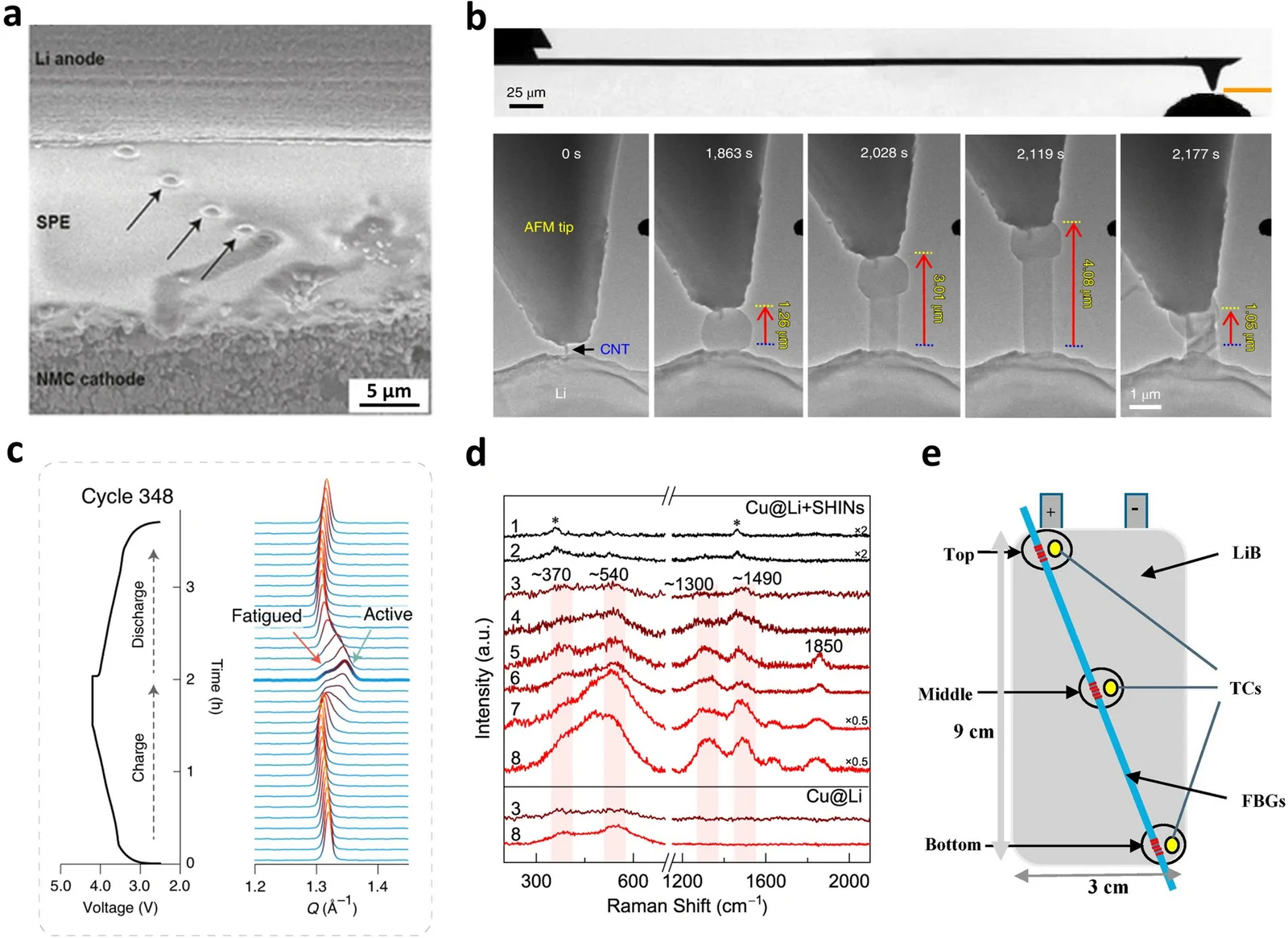
*In situ/operando* studies of lithium batteries by various characterization techniques. **a** Degradation of SPE as a function of time is shown in secondary electron images. Reproduced with permission from Ref. [178]. Copyright 2020, American Chemical Society. **b** AFM-ETEM characterization of stress generation during Li whisker growth. Reproduced with permission from Ref. [180]. Copyright 2020, Springer Nature. **c** *Operando* long-duration SR-PXRD investigation of an NMC811/graphite full cell. Reproduced with permission from Ref. [183]. Copyright 2020, Springer Nature. **d** The formation and evolution of SEIs in the dual-salt electrolyte revealed by DS-PERS. Reproduced with permission from Ref. [187]. Copyright 2023, Springer Nature. **e** (left) Schematic of rechargeable LiB test setup with the location of TCs and FBGs, and (right) schematic diagram. Reproduced with permission from Ref. [192]. Copyright 2017, Elsevier Ltd

Lithium-ion batteries dominate portable electrochemical energy storage, with cathode material stability and lithium storage voltage critically determining energy and power density. Recent research has focused on understanding structural changes during cycling, coating, and doping using advanced imaging techniques. Xiao and co-workers have conducted extensive and influential research on developing highly stable lithium-ion cathode materials and elucidating their mechanisms using TEM [175, 176]. For instance, they developed an ionic liquid (IL)-assisted two-step synthesis method to fabricate a NiO/nitrogen-doped carbon (NiO/NDC) composite with a hierarchical hollow spherical architecture. This structure effectively mitigates issues such as volume expansion and poor electrical conductivity when NiO is used as an anode material in lithium-ion batteries [177]. Using *in situ* TEM, they monitored in real-time the morphological evolution and phase transformation of the hollow microstructure, directly demonstrating the material’s structural thermal stability. This work provides a promising strategy for the development of anode materials for high-performance lithium-ion batteries (LIBs). Zaghib et al. used *in situ* SEM to observe the degradation processes of solid polymer electrolyte (SPE) under the nickel-manganese-cobalt oxide (NMC) cathode in an all-solid-state lithium-ion polymer battery (Fig. 14a) [178]. Qin et al. used *in situ* LSCM to observe the dynamic morphology of the lithium metal surface and quantified dendrite suppression via 3D/2D imaging [179]. Combined with electrochemical performance such as 700-h cycling stability, their results clearly demonstrate the interfacial optimization advantages of DC-SPE. It was found that the main mechanism of SPE degradation is chemical degradation, manifested as the gradual thinning of SPE and the generation of gas. Huang et al. used the *in situ* atomic force microscopy-environmental transmission electron microscopy (AFM-ETEM) technique to study the growth mode and morphological change rules of lithium dendrites, as well as the mechanism and influencing factors of stress generation [180]. As shown in Fig. 14b, through *in situ* observation and stress measurement, it was found that the dendrite growth and stress generation processes of lithium metal in solid electrolytes are different from those in liquid electrolytes. The dendrite growth rate of lithium metal in solid electrolytes is slower, the morphology is more regular, and the mechanism of stress generation is also different. Meng et al. employed *in situ* EC-AFM-Raman to characterize the charge–discharge process of LiMn_2_O_4_ [181]. AFM revealed particle expansion and contraction during lithiation and delithiation, while Raman spectroscopy detected spectral shifts indicative of phase transitions. This study demonstrates the utility of multimodal techniques in probing battery materials.

The morphological, structural, and compositional evolution of electrode materials during charge–discharge processes is critical to bridging initial electrode design and ultimate electrochemical performance [182]. Understanding these evolution patterns and establishing a correlation among structural dynamics, reaction kinetics, and performance is essential for advancing battery materials research. *In situ* XRD remains a powerful tool for tracking phase and lattice parameter changes during cycling. Grey et al. used the *in situ* synchrotron radiation X-ray diffraction (SR-PXRD) technique to study the structural changes of NMC811 during the charging and discharging processes, and discovered the formation and evolution of a fatigue phase (Fig. 14c) [183]. The experimental results show that NMC811 undergoes surface reconstruction at a high state of charge, forming a surface layer with a rock salt structure. This surface reconstruction causes a mismatch in the bulk structure, which leads to bulk fatigue. XPS provides surface composition, valence states, and energy-level information. Masuda et al. used *in situ* XPS to study the lithiation and delithiation reactions of silicon thin-film electrodes in all-solid-state lithium-ion batteries [184]. During the lithiation processes, lithium silicide and lithium silicate are formed on the surface of the silicon electrode, and during the delithiation processes, lithium silicide and lithium silicate are partially decomposed, while lithium oxide and lithium carbonate still exist. Liu et al. systematically investigated the size-dependent fracture behavior of silicon nanoparticles (SiNPs) during initial lithiation using *in situ* TEM, complemented by electrochemical/chemical lithiation experiments and stress simulations [185]. Their work revealed that surface cracking originates from stress reversal at the two-phase interface during lithiation, while smaller particles avoid fracture due to insufficient strain-energy release.

The solid electrolyte interphase (SEI) is a passivation layer formed at the electrode/electrolyte interface during the initial charging cycle, enabling ion conduction while blocking electron transfer. A stable SEI is critical for enhancing the cycle life and safety of lithium-ion batteries (LIBs). Its morphology and structure vary with electrolyte composition, significantly impacting battery performance. Understanding the structure–activity relationship of SEI is thus essential for optimizing LIBs. Wan et al. used *in situ* AFM to monitor the SEI film formation and lithium-ion intercalation/deintercalation process on ultrathin monolayer molybdenum disulfide [186]. The study showed that a network of wrinkled structures formed on the surface of molybdenum disulfide during the lithiation processes, which is related to the inherent flexibility of the material and the failure mechanism of the battery. Mao et al. employed dynamic surface-enhanced Raman spectroscopy (DS-SERS) and theoretical calculations to analyze SEI formation [187]. They revealed that SEI evolution is closely tied to Li-ion desolvation and deposition (Fig. 14d). Li^+^ desolvates at the SEI/electrolyte interface, diffuses through the SEI, and reacts at the metal/SEI interface, influencing SEI composition and structure. The study also highlighted the role of electrolyte composition and concentration in SEI properties, offering valuable insights for designing high-performance LIBs. The high Li⁺ desolvation energy barrier at the cathode-electrolyte interface (CEI) contributes to sluggish charge transfer, which in turn limits power density and low-temperature performance. Lu et al. tackled the “stability-kinetics” trade-off at the CEI by disrupting the molecular symmetry of conventional symmetric sulfonimides (e.g., commercial LiTFSI), designing and synthesizing two novel lithium sulfinimide salts [188]. Using liquid chromatography–quadrupole time-of-flight mass spectrometry (LC-QTOF-MS), they captured anionic polymerization intermediates, elucidating the “oxidation-polymerization” pathway of STFSI⁻. This work provides molecular-level insight into cathode interfacial engineering.

The service life and safety of lithium batteries have always been of great concern to customers. Advanced sensor technology can monitor the physical and chemical signals of lithium batteries in real time and accurately, providing a basis for battery condition assessment and safety warnings. Rechargeable lithium batteries (RLBs) typically perform poorly under extreme temperatures, necessitating strategies to enhance their temperature tolerance for diverse applications [189]. Additionally, thermal runaway during charging/discharging poses significant safety risks, including combustion or explosion [190]. Effective battery thermal management systems (BTMS) are essential to monitor and control temperature changes. For example, by embedding K-type micro-thermocouples in experimental lithium-ion batteries, Huang et al. were able to measure the temperature distribution within the battery during internal short circuits and thermal runaway [191]. During internal short circuits and thermal runaway, the observed temperature distribution exhibited a high degree of non-uniformity compared to constant current discharge and external short circuits. Pinto’s group compared the responses of thermocouples and fiber Bragg grating sensors (FBGs) when monitoring temperature changes at different locations in lithium batteries (Fig. 14e) [192]. It was found that FBGs had better performance in monitoring the surface temperature of the battery than thermocouples, and could respond more accurately to temperature changes in the battery under heavy-duty cycling. They also used an FBG sensor network to perform real-time, *in situ* multi-point monitoring of temperature mapping in lithium polymer battery packs [193]. It is highlighted that FBG sensing networks can be used to improve the thermal management of batteries by performing spatiotemporal thermal mapping and identifying areas that are more prone to hotspots, thereby preventing serious consequences such as thermal runaways and promoting battery safety.

Solid-state lithium batteries (SSBs) promise enhanced safety, energy density, and power density, but their mechanical behavior during operation remains underexplored. Zhang et al. used a self-made device (hot-press setup) combined with hydraulic pressure equipment and an electronic pressure gauge to monitor the pressure and height changes of the battery in real-time during the charging and discharging processes [194]. It was found that the rigidity of the solid electrolyte results in significant pressure changes during the charging and discharging processes, which has a significant impact on battery performance, and the use of zero-strain anode materials can reduce this effect.

### Li-sulfur/Li-oxygen Batteries

Compared with lithium-ion batteries, the specific energies of Li–S and Li-O_2_ batteries have increased significantly, and have received extensive attention from scientific researchers. However, problems such as slow redox kinetics, severe shuttle effects [195], electrolyte depletion, and degradation of the lithium anode still hinder commercialization. Studying the basic reaction mechanisms of each component in the system is crucial to solving the above problems and further improving the overall performance of the battery.

**Fig. 15:**
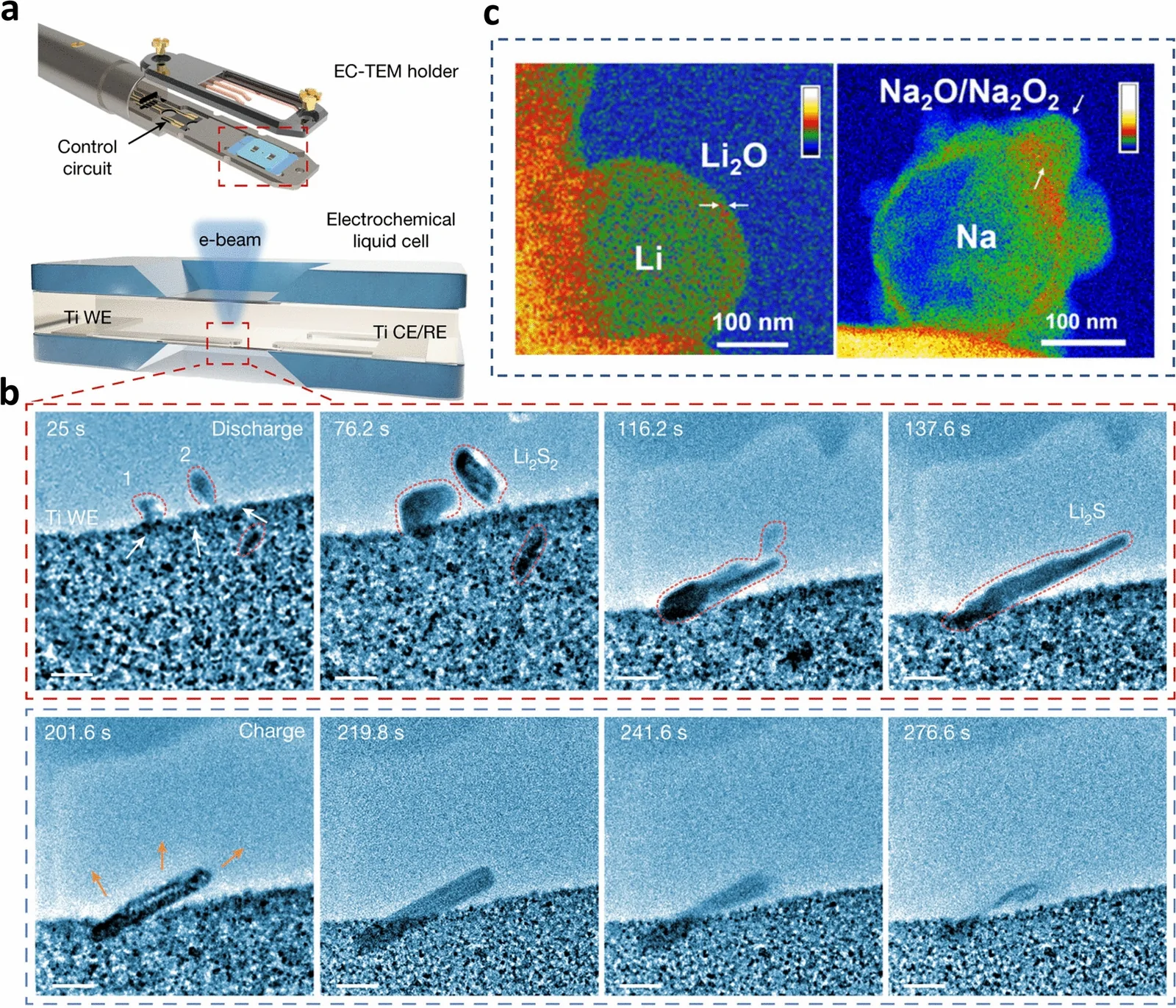
*In situ/operando* studies of Li-sulfur/Li-oxygen batteries by TEM characterization techniques. **a** Configuration of liquid-cell EC-TEM and a schematic illustration of electrochemical reactions of LiPSs at different electrode–electrolyte interfaces. **b** Time-series TEM images of Li_2_S deposition (dashed red frame) and dissolution (dashed blue frame) in an electrochemical liquid cell. Reproduced with permission from Ref. [196]. Copyright 2023, UChicago Argonne, LLC, Operator of Argonne National Laboratory. **c** Time-lapse TEM images of Li and Na in dry air. Reproduced with permission from Ref. [197]. Copyright 2023, American Chemical Society

Microscopic imaging techniques are pivotal for studying electrochemical and failure mechanisms during battery cycling. Liao et al. achieved real-time and high-resolution transformation processes of LiPSs on the electrode surface by constructing Li–S nano-batteries based on ether electrolyte and combining them with electrochemical transmission electron microscopy technology (Fig. 15a) [196]. Without the participation of active centers, LiPSs follow the traditional single-molecule path and gradually convert to Li_2_S_2_ and Li_2_S. In the presence of active centers, LiPSs will accumulate on the surface of the active center to form a high-density ionic complex phase, thereby inducing the instantaneous deposition of Li_2_S (Fig. 15b). The discovery of the collective reaction path provides a new perspective for understanding the reaction mechanism of Li–S batteries. Hu et al. first used *in situ* environmental transmission electron microscopy (ETEM) combined with theoretical simulations to reveal the different stabilities of Li and Na in dry air (Fig. 15c) [197]. The study found that a dense Li_2_O layer was formed on the Li surface, while a porous and rough Na_2_O/Na_2_O_2_ layer was formed on the Na surface, which was due to the thermodynamic and kinetic differences of O_2_ on the Li and Na surfaces. Lee et al. characterized the structure and chemical composition of the adaptive protective layer (APL) model by SEM and XPS, and the results showed that the APL model consists of an inner layer of high surface energy polymer (PEO) and an outer layer of high modulus polymer (PVDF-HFP), which can effectively suppress the diffusion of LiPS and the decomposition of the electrolyte, and improve the cycle life of the battery [198]. Wan et al. installed an AFM probe inside the battery to observe the interface changes of the battery in real-time during the charging and discharging processes [199]. *In situ* AFM observations revealed that the introduction of water changed the nucleation pathway of Li_2_O_2_ from a surface-mediated mechanism to a solution-mediated mechanism.

*In situ* XANES is indispensable for rapid, high-precision analysis of element valence states and distributions in solid-state batteries, offering non-destructive insights into charge/discharge and failure mechanisms. Hu et al. monitored the concentration changes of PS and its products at different charge and discharge stages of the battery by *operando* sulfur K-edge X-ray absorption spectroscopy, combined with theoretical calculations, which revealed the reaction processes and products of LiNO_3_ and PS, which is related to the concentration gradient of PS [200]. It shows that the role of LiNO_3_ is to oxidize PS to a higher valence product, thereby stabilizing the battery. This study provides a new perspective for understanding the mechanism of LiNO_3_ in Li–S batteries and provides theoretical guidance for designing a more stable Li–S battery. Lithium dendrite penetration through ceramic electrolytes remains a major challenge for high-energy–density solid-state batteries. Bruce’s group employed *in situ* X-ray computed tomography combined with spatially mapped X-ray diffraction to track the propagation and spread of cracks and lithium dendrites within the ceramic electrolyte in a Li/Li_6_PS_5_Cl/Li cell [201]. Their findings revealed that during the dendrite growth processes in all-solid-state batteries, cracks propagate through the ceramic far ahead of the metallic lithium. During lithium deposition, spallation forms in the electrolyte adjacent to the deposition electrode, propagating along paths with higher porosity than the average of the ceramic toward the deposition electrode surface. Based on the spallation, deep cracks penetrate the entire solid electrolyte, creating a pathway between the deposition and stripping electrodes without initially forming a short circuit. Ultimately, when the deposited lithium metal (lithium dendrites) grow and fill the cracks to reach the stripping electrode, it finally causes a battery short circuit.

**Fig. 16:**
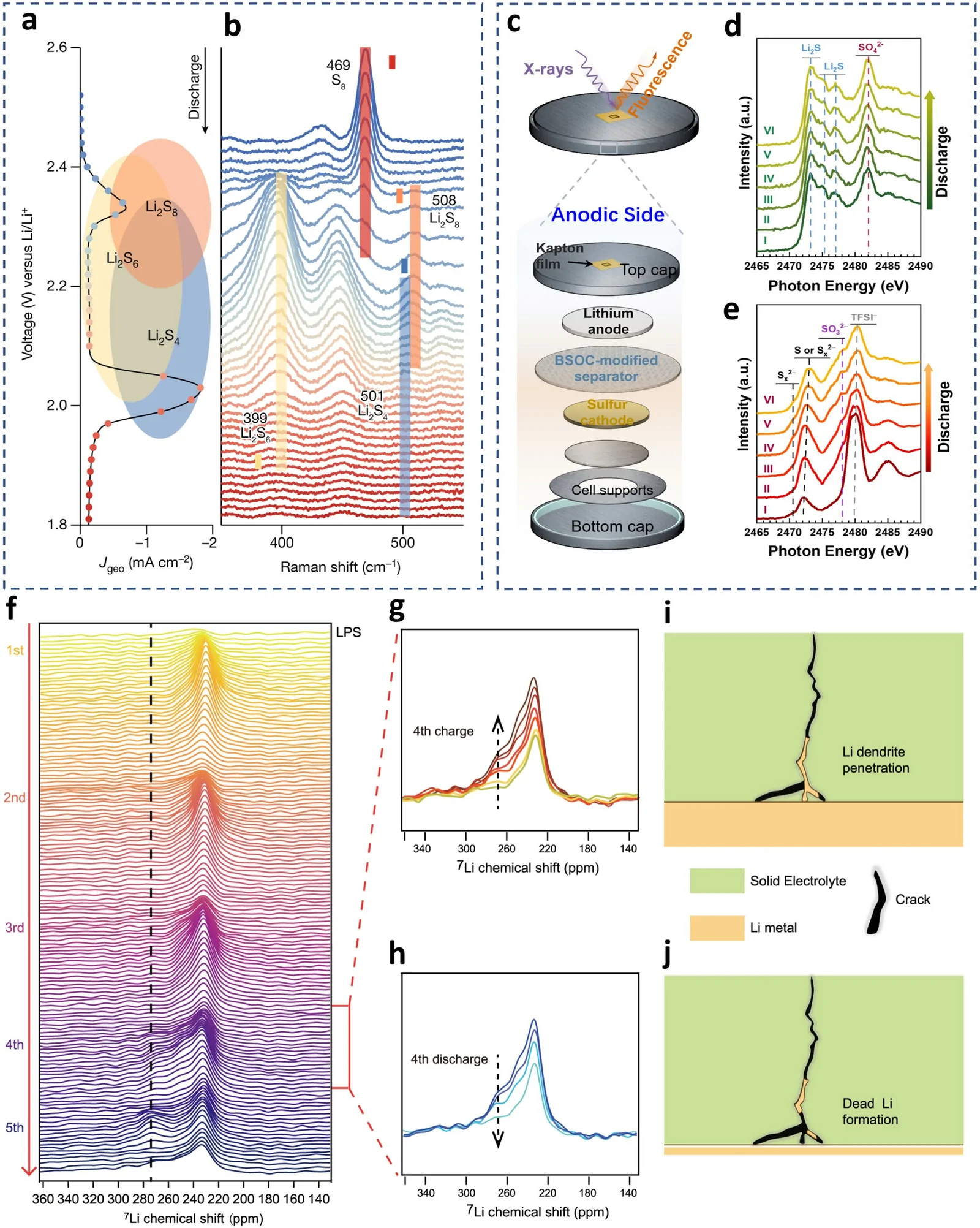
*In situ/operando* studies of Li-sulfur/Li-oxygen batteries by spectroscopy characterization techniques. CV profile (**a**) and experimental *in situ* Raman spectra (**b**), with colors corresponding to voltages. The Raman cell was run with a discharge CV scan at 0.05 mV s^−1^ when data were being collected. Characteristic peaks used to quantify intermediates are marked in corresponding color shades. Small labels with a darker color indicate computed frequency values. Raman results during discharge with the N, S-HGF catalytic electrode. Reproduced with permission from Ref. [202]. Copyright 2024, Springer Nature. **c** Schematic illustration of the coin cell design for AEI observation in *in situ*/*operando* XAS study. **d** *in situ* representative XAS spectra collected at different potentials for Li/S cell with a PP separator during the first discharge process. **e** *in situ* representative XAS spectra collected at different potentials for Li/S cell with BSOC-PP separator during the first discharge process. Reproduced with permission from Ref. [205]. Copyright 2020, Zhengzhou University. *Operando* 7Li NMR stack spectra of AFB (with LPS) during the first five cycles (**f**), with magnified stacking spectra of the 4th charging process (**g**) and the 4th discharging process (**h**). Schematic of the lithium dendrite penetration process (**i**) and dead Li formation after subsequent discharging (**j**). Reproduced with permission from Ref. [206]. Copyright 2023, Springer Nature

Understanding the redox reactions in lithium-sulfur batteries is critical for improving capacity and kinetics. Duan et al. used *in situ* Raman spectroscopy to systematically study the reaction network of electrocatalytic SRR and identified key intermediate products (S_8_, Li_2_S_8_, Li_2_S_6_, Li_2_S_4,_ and Li_2_S) and the main reaction path (Fig. 16a, b) [202]. It was found that Li_2_S_4_ is the key electrochemical intermediate that controls the kinetics of the entire SRR, while Li_2_S_6_ is mainly generated or consumed through non-electrochemical recombination/decomposition reactions. Zhang et al. conducted an in-depth study on the regulatory effect of cobalt phthalocyanine (CoPc) on the solvolytic dissociation behavior of lithium polysulfides in ether-based solvents and its inhibitory effect on the shuttle phenomenon [203]. *In situ* Raman experiments demonstrated that CoPc can promote the dissociation equilibrium of lithium polysulfides (LiPSs) toward the generation of more sulfide anions through interaction with these anions. Consequently, the production of Li-LiPSs^+^ cations is reduced, leading to a corresponding suppression of the shuttle effect. Wang et al. found that the Ni@C/graphene composite can effectively suppress the shuttle effect of polysulfides and improve the cycle stability and rate performance of the battery [204]. *In situ* ATR FT-IR and DFT calculation results show that the Ni@C/graphene composite can promote the redox kinetics of polysulfides, making them convert to Li_2_S faster. By introducing a bismuth sulfide/bismuth oxide nanocluster in a carbon matrix (BSOC) electrocatalytic layer to modify the separator, the cycle performance and rate performance of Li/S batteries were significantly improved. It was found that the BSOC electrocatalytic layer can promote the transformation and utilization of polysulfides, thereby improving the capacity and cycle life of the battery. Zhang et al. monitored the chemical state and distribution of polysulfides in Li/S batteries during charge and discharge in real-time by *in situ*/*operando* XAS technology, as shown in Fig. 16c [205]. It was found that during charging, polysulfides shuttle between the cathode-electrolyte-separator interface (CESI) and the electrolyte-anode interface (AEI), resulting in negative electrode corrosion and battery performance degradation (Fig. 16d, e). The BSOC electrocatalytic layer can effectively capture and convert polysulfides, thereby suppressing the shuttle effect of polysulfides. Yang and co-workers employed *in situ* NMR to achieve quantitative tracking and morphological correlation of dead Li and SEI-incorporated Li [206]. Backscattered electron (BSE) imaging showed dark regions within the solid-state electrolytes (SSEs), with corresponding Li signals detected by NMR (Fig. 16f-h). This was attributed to the formation of electronically isolated dead Li after Li dendrites penetrated SSE cracks and lost electrical contact with the current collector during stripping (Fig. 16i, j).

The crossover of oxygen from the cathode to the anode is an inevitable phenomenon in most Li-O_2_ batteries, and its impact on the formation and operation of the SEI on the lithium metal anode (LMA) remains insufficiently explored. Peng’s group reported the influence of oxygen on SEI formation in Li-O_2_ batteries with a model Cu/DMSO interface [207]. *In situ* SERS and FT-IR experiments demonstrated that oxygen can inhibit the cleavage of the C-S bond in the DMSO solvent, thereby reducing the formation of unstable SEI components (such as C≡C species) and volatile products (such as C_2_H_6_ and H_2_). As a result, the SEI formed under oxygen is more uniform, with fewer voids, and improves the electrochemical performance of the LMA. This work provides new insights into the crossover effects of oxygen on SEI chemistry, which is beneficial for designing better LMA/electrolyte interfaces for future Li-O_2_ batteries. The electrochemical deposition behavior of insulating and insoluble products, such as lithium peroxide (Li_2_O_2_) and lithium carbonate (Li_2_CO_3_), generated on the cathode surface of non-aqueous lithium-oxygen batteries has not been systematically understood. Recently, Ye’s group utilized *in situ* SERS to probe the competitive changes of solvents and products within the interfacial region of a gold electrode [208]. By monitoring the adsorption signals of DMSO solvent molecules (υ(Au–S)) and the intermediate Li-O_2_ (υ(Au-O)) on the electrode surface, the spontaneous desorption behavior of the reaction product Li_2_O_2_ was verified under both electrochemical *in situ* reaction conditions and open-circuit potential (OCP). The authors proposed that the desorption driving force of the insulating deposition products originates from interfacial solvation repulsion, which continuously releases active sites on the electrode surface, thereby ensuring that the ORR always occurs at the electrode/Li_2_O_2_ interface.

## Conclusions and Outlook

In summary, *in situ* studies of EECSTs encompass diverse approaches, such as characterizing electrode material evolution, monitoring intermediates, detecting products, and sensing surroundings. By probing chemical/electrochemical processes at the atomic and molecular level, these techniques provide critical insights into complex reaction mechanisms. Despite considerable progress, challenges persist in enhancing sensitivity, spatial resolution, and applicability to complex interfaces or large-scale systems. To overcome these limitations, we propose the following future research directions:

1. Ultra-high temporal and spatial resolution: A key trend in *in situ* studies involves enhancing temporal resolution to capture short-lived intermediates and ultrafast kinetic processes. For instance, advances in fast-scanning and ultrafast spectroscopic techniques enable real-time monitoring of electrochemical dynamics, offering direct evidence of transient intermediates and excited states. Simultaneously, improving spatial resolution is essential for elucidating electrochemical mechanisms at the microscopic level. Techniques such as high-resolution AFM/STM and synchrotron radiation-based spectroscopy can reveal fine structural details and dynamic changes of adsorbed molecules on electrode surfaces. The advancement of *in situ* characterization methods with ultra-high spatiotemporal resolution is therefore critical for obtaining an accurate and detailed understanding of electrochemical behavior.
2. Multi-technique combination: Electrochemical reactions entail complex interactions among multiple active structures, relying solely on a single in situ technique for monitoring and mechanistic interpretation may lead to biased conclusions. Therefore, it is essential to integrate multiple in situ characterization methods. For instance, coupling in situ spectroscopy with microscopic imaging can provide comprehensive insights into the electrode/electrolyte interface, including morphology, composition, electronic structures, and reaction intermediates. Furthermore, multi-technique approaches capture reaction dynamics across time scales (such as combining SERS with electrochemical noise analysis, or integrating liquid-cell TEM with ultrafast techniques), facilitating a multidimensional understanding of structure-performance correlations and complex reaction mechanisms. Integrating multiple techniques highly into a single system to form a “multimodal in situ characterization functional island” represents the current development trend.
3. Operando characterizations: Many electrode materials demonstrating excellent performance in half-cell tests fail in commercial applications, primarily due to the disparity between laboratory-scale electrochemical cells and actual devices. Advancing in situ characterization from simulated environments to real application scenarios (such as batteries, fuel cells, and water electrolyzers etc.) constitutes a critical direction for next-generation research. Such s transition provides electrochemical insights closer to practical conditions, guiding the optimization of electrochemical systems. Through the design of operando experimental devices, it becomes possible to mimic industrial reaction conditions and achieve truly relevant in situ measurements.
4. Artificial intelligence: The application of artificial intelligence (AI) technology significantly enhances the efficiency of data analysis in electrochemical research. Through machine learning and data mining, valuable insights can be extracted from complex datasets. AI algorithms facilitate the prediction of electrochemical processes, optimization of experimental conditions, molecular dynamics modeling, and even the design of novel electrodes. For instance, the integration of highly sensitive in situ/operando spectroscopic techniques (such as enhanced Raman spectroscopy, infrared spectroscopy, mass spectrometry, fiber optic sensing, and X-ray methods) with AI-enabled data interpretation enables a closed-loop research paradigm: AI-assisted “dynamic characterization-interpretation feedback-precise control”.

As these advancements mature, *in situ* characterization is poised to evolve from a diagnostic tool into a predictive platform that can inform the rational design of next-generation batteries, electrocatalysts, and renewable energy systems. Ultimately, this review advances the understanding and application of *in situ* characterization, underscores its transformative potential, and provides a roadmap for integration of these methodologies as foundational technologies in sustainable energy research. These approaches provide critical insights into the fundamental mechanisms of key electrochemical processes, enable the establishment of accurate structure–property relationships, and guide the design of more efficient and stable energy materials and devices.
